# The Prevalence of Symptomatic Dermographism: Results of the International UCARE PREVALENCE‐D Study

**DOI:** 10.1111/all.70047

**Published:** 2025-09-13

**Authors:** Kanokvalai Kulthanan, Jonathan A. Bernstein, Michael Rudenko, Pascale Salameh, Chulaluk Komoltri, Esra Adışen, Salma Al Abri, Mona Al‐Ahmad, Nasser Al‐Ahmed, Bushra Al Hinai, Anastasiia Allenova, Saad Alshareef, Nattha Angkoolpakdeekul, Rand Arnaout, Joanna Bartosińska, Ivan Cherrez‐Ojeda, Leena Chularojanamontri, Paulo Ricardo Criado, Luis Felipe Ensina, Roberta Fachini Jardim Criado, Cesar Alberto Galvan Calle, Ana Maria Giménez‐Arnau, Kiran Godse, Maia Gotua, Naoko Inomata, Nuttagarn Jantanapornchai, Chang‐Gyu Jung, Alicja Kasperska‐Zając, Maryam Khoshkhui, Pavel Kolkhir, Dorota Krasowska, Jomgriditip Laomoleethorn, Antonina Maiorowa, Raisa Meshkova, Dragan Mijakoski, Melba Munoz, Yanisorn Nanchaipruek, Iman Nasr, Rabia Öztaş Kara, Waratchaya Panjapakkul, Teerapat Paringkarn, Indrashis Podder, Karla Robles‐Velasco, Isabel Rosmaninho, Ana Rita Presa, Chuda Rujitharanawong, Phuwakorn Saengthong‐aram, Rana Tafrishi, Natasa Teovska Mitrevska, Papapit Tuchinda, Teerapat Wannawittayapa, Anushka Wilson, Young‐Min Ye, Anna Zalewska‐Janowska, Marcus Maurer, Torsten Zuberbier

**Affiliations:** ^1^ Department of Dermatology, Faculty of Medicine Siriraj Hospital Mahidol University Bangkok Thailand; ^2^ Division of Rheumatology, Allergy and Immunology University of Cincinnati College of Medicine Cincinnati Ohio USA; ^3^ London Allergy and Immunology Centre London UK; ^4^ Institute of Allergology, Charité—Universitätsmedizin Berlin Corporate Member of Freie Universität Berlin and Humboldt‐Universität Zu Berlin Berlin Germany; ^5^ Fraunhofer Institute for Translational Medicine and Pharmacology ITMP, Immunology and Allergology Berlin Germany; ^6^ Gilbert and Rose‐Marie Chagoury School of Medicine Lebanese American University Beirut Lebanon; ^7^ Department of Primary Care and Population Health University of Nicosia Medical School Nicosia Cyprus; ^8^ Faculty of Pharmacy Lebanese University Beirut Lebanon; ^9^ Institut National de Santé Publique D'épidémiologie Clinique et de Toxicologie‐Liban (INSPECT‐LB) Beirut Lebanon; ^10^ Division of Research and Development, Faculty of Medicine Siriraj Hospital Mahidol University Bangkok Thailand; ^11^ Department of Dermatology, Faculty of Medicine Gazi University Ankara Turkey; ^12^ Clinical Immunology and Allergy Unit Royal Hospital Muscat Oman; ^13^ College of Medicine Kuwait University Kuwait City Kuwait; ^14^ Al‐Rashed Allergy Center Ministry of Health Kuwait City Kuwait; ^15^ Laboratory of Immune‐Mediated Skin Diseases Institute of Regenerative Medicine, Biomedical Science & Technology Park, I.M. Sechenov First Moscow State Medical University (Sechenov University) Moscow Russian Federation; ^16^ King Faisal Specialist Hospital and Research Center Riyadh Saudi Arabia; ^17^ Department of Cosmetology and Aesthetic Medicine Medical University of Lublin Lublin Poland; ^18^ Department of Dermatology, Venereology and Pediatric Dermatology Medical University of Lublin Lubin Poland; ^19^ Universidad Espíritu Santo Samborondón Ecuador; ^20^ Respiralab Research Group Guayaquil Ecuador; ^21^ Department of Dermatology Centro Universitário Faculdade de Medicina do ABC Santo André Brazil; ^22^ Federal University of São Paulo São Paulo Brazil; ^23^ Emedic Salud Lima Peru; ^24^ Department of Dermatology, Hospital del Mar Research Institute Universitat Pompeu Fabra Barcelona Spain; ^25^ Department of Dermatology D. Y. Patil University and School of Medicine Navi Mumbai India; ^26^ Center of Allergy and Immunology David Tvildiani Medical University Tbilisi Georgia; ^27^ Department of Dermatology Showa University School of Medicine Tokyo Japan; ^28^ Department of Allergy and Clinical Immunology Keimyung University School of Medicine Daegu South Korea; ^29^ Department of Clinical Allergology and Urticaria Medical University of Silesia Katowice Poland; ^30^ Allergy Research Center Mashhad University of Medical Sciences Mashhad Iran; ^31^ Smolensk State Medical University Smolensk Russia; ^32^ Institute of Occupational Health of R.N. Macedonia WHO Collaborating Center, GA^2^LEN Collaborating Center Skopje Republic of North Macedonia; ^33^ Faculty of Medicine, Ss. Cyril and Methodius University of Skopje Skopje Republic of North Macedonia; ^34^ Department of Dermatology Sakarya University Faculty of Medicine Sakarya Turkey; ^35^ Department of Dermatology College of Medicine and Sagore Dutta Hospital Kolkata India; ^36^ Unidade Local de Saúde Gaia e Espinho Vila Nova de Gaia Portugal; ^37^ Tâmega/Sousa Hospital Center Penafiel Portugal; ^38^ Dermatology Department Remedika General Hospital Skopje Republic of North Macedonia; ^39^ Department of Dermatology International Balkan University Skopje Republic of North Macedonia; ^40^ Department of Allergy and Clinical Immunology Ajou University School of Medicine Suwon South Korea; ^41^ Psychodermatology Department Medical University of Lodz Lodz Poland

**Keywords:** chronic inducible urticaria, dermographism, prevalence, symptomatic dermographism, urticaria

## Abstract

**Background:**

The prevalence of symptomatic dermographism (SD) in the general population remains unclear. This study aimed to internationally estimate the prevalence of SD and two other dermographic subtypes—physiological red dermographism and simple urticarial dermographism—among adults worldwide.

**Methods:**

The PREVALENCE‐D (Prevalence Estimation of Dermographism) study was the largest international, cross‐sectional, internet‐based investigation conducted by the Urticaria Centers of Reference and Excellence of the Global Allergy and Asthma Excellence Network from 2021 to 2024. A world expert‐designed questionnaire was completed by participants from 28 centers across 19 countries to diagnose SD. Participants were recruited into three age groups (18–24, 25–60, and > 60 years), with ≥ 1000 individuals per group per country. We calculated sex‐ and age‐adjusted prevalence estimates internationally and by country, with 95% confidence intervals.

**Results:**

Among 59,543 participants worldwide, those with SD had an adjusted point prevalence of 3.20% (95% confidence interval [CI] 2.68%–3.73%) and a lifetime prevalence of 5.94% (95% CI 5.32%–6.56%). The 25–60 years group presented the highest prevalence. Females had higher rates than males, with statistically significant differences across all age groups for both point and lifetime prevalence. Physiological red dermographism had an adjusted point prevalence of 10.02% (95% CI 9.48%–10.55%) and a lifetime prevalence of 33.47% (95% CI 32.01%–34.94%). Simple urticarial dermographism showed an adjusted point prevalence of 1.21% (95% CI 1.08%–1.35%) and a lifetime prevalence of 5.39% (95% CI 4.99%–5.79%).

**Conclusion:**

These findings provide comprehensive international estimates of dermographism, particularly SD, and emphasize the need for appropriate healthcare resource allocation and disease recognition.

## Graphical abstract

An online expert‐designed questionnaire estimated the international prevalence of dermographism. Symptomatic dermographism, physiological red dermographism, and simple urticarial dermographism exhibit distinct prevalence patterns across age groups, sex, and geographic regions. Symptomatic dermographism has an international point prevalence of 3.20% (95% CI 2.68%–3.73%) and a lifetime prevalence of 5.94% (95% CI 5.32%–6.56%), with significantly higher rates in females and the working‐age group. Abbreviations: CI, confidence interval; ns, non‐significant.

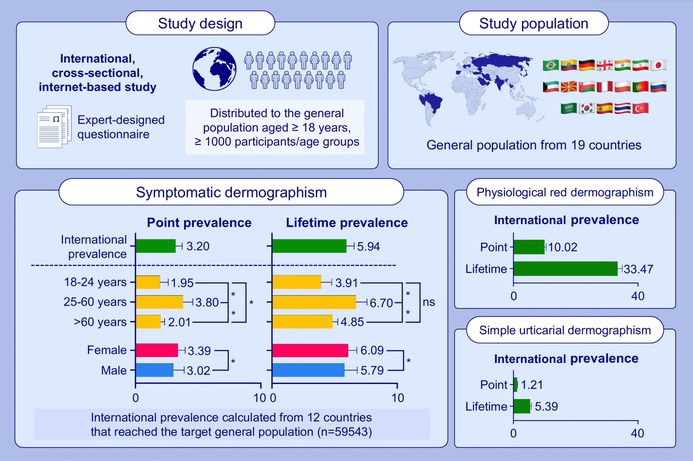

## 1 Introduction

Symptomatic dermographism (SD), formerly referred to as urticaria factitia or dermographic urticaria, is the most common form of chronic inducible urticaria. It is mostly characterized by strip‐shaped, pruritic wheals triggered by skin friction, such as stroking, scratching, scrubbing, or rubbing, but it can also be seen as flatter and broader wheals if the area of friction is larger, for example, on the palms when using tools like a screwdriver. These wheals typically appear within 1 to 5 min and persist for approximately 30 min [1, 2]. Many patients with SD experience pruritic episodes for years [3, 4], which substantially impairs quality of life, including psychological stress and ability to work, particularly in occupations with chronic shearing forces on the palms and feet, restricts daily activities, and necessitates medical attention [4, 5, 6]. The exact prevalence of SD remains unclear, with estimates ranging from 0.1% to 5.0% [4, 7, 8, 9, 10, 11, 12, 13, 14, 15].

**FIGURE 1:**
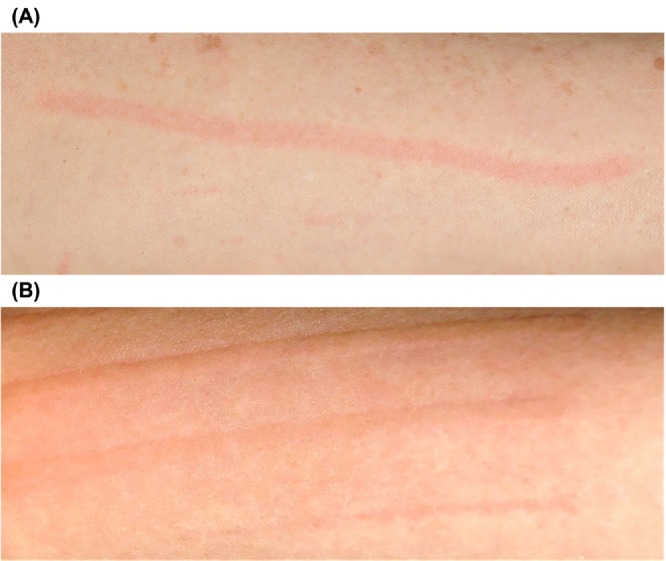
Clinical manifestations of each type of dermographism. (A) Physiological red dermographism. (B) Urticarial dermographism (If wheals are not itchy, they are classified as simple urticarial dermographism. Individuals with recurrent, itchy urticarial dermographism are diagnosed with symptomatic dermographism).

Simple urticarial dermographism (SimUD) and physiological red dermographism (PD) differ from SD in that neither involves itching. In PD, scratching causes transient erythema but without wheals (Figure 1A), whereas SimUD is characterized by scratching‐induced wheals (Figure 1B). To date, there is no consensus on the definition of PD. However, in 1924, Lewis described a transient cutaneous reaction known as the triple response of Lewis, which occurs following firm stroking of the skin. This results in an initial transient erythematous line, which is quickly followed by a broader area of erythema and subsequently a surrounding flare. This sequence represents a normal vascular response to frictional stimulation of the skin [11]. Warin [16] and Wong et al. [17] defined red dermographism as a cutaneous response in which rubbing initially induces erythema in the surrounding skin, shortly followed by a diffuse wheal within that erythematous area, whereas scratching induces only transient erythematous streaks. As with SD, little is known about the prevalence of PD and SimUD.

In 2016, four major dermatological and allergy organizations reached a consensus on chronic inducible urticaria [1]. According to this consensus, SD is a physical urticaria defined by (i) friction‐induced wheals that are symptomatic and (ii) a positive provocation test. Provocation testing is done by firm stroking of the skin of the volar forearm or upper back with a smooth blunt object (e.g., closed ballpoint pen or a wooden spatula) or with a dermographometer (e.g., a dermographic tester [HTZ Ltd., New Addington, United Kingdom] or FricTest [Moxie, Berlin, Germany]). A dermographometer provides standardized and reproducible test settings and secures the application of appropriate trigger strength, such as stroking of the skin with the dermographic tester at a pressure of 36 g/mm^2^. It also enables the testing of individual trigger thresholds, an important measure of SD disease activity [1, 18, 19]. The skin response is evaluated after 10 min and is considered positive if a wheal appears at the provocation site and elicits pruritus or a burning sensation [1].

Many studies have investigated the prevalence of SD and related subtypes (Table S1). However, small sample sizes, a lack of general population representation, and inconsistent definitions of dermographism subtypes limit conclusive findings. In addition, data on how prevalence varies by sex, country, or age group are scarce. Epidemiological efforts should be undertaken worldwide to identify geographic, sex‐based, and age‐related differences—similar to those reported for chronic spontaneous urticaria [20]. Clarifying the prevalence of SD in the general population is essential for planning healthcare resource allocation, supporting clinical trials, and detecting trends in follow‐up studies.

To address these knowledge gaps, we conducted the PREVALENCE‐D (Prevalence Estimation of Dermographism) study at the Urticaria Centers of Reference and Excellence (UCAREs) of the Global Allergy and Asthma Excellence Network. Since there is no consensus definition for red dermographism, with controversy remaining, the steering committee members—all of whom were UCARE members and global experts in urticaria—proposed questions in this survey questionnaire to categorize cutaneous response to firm stroking into three categories as follows: (i) PD: a transient erythematous line, without wheal or itch; (ii) SimUD: wheal formation without itch; and (iii) SD: wheal formation accompanied by pruritus or burning.

Our primary objective was to determine the overall prevalence of SD in participants aged 18 years or older from geographically diverse regions via an internet‐based survey. Our secondary objectives were to (i) investigate the prevalence of PD and SimUD; (ii) stratify SD, PD, and SimUD prevalence by age; (iii) stratify them by sex; and (iv) compare SD prevalence between females and males.

## 2 Methods

### 2.1 Study Design and Conduct

PREVALENCE‐D was an international, cross‐sectional study performed by the UCAREs of the Global Allergy and Asthma Excellence Network from December 2021 to December 2024. It involved 28 centers across 19 countries. The study received ethical approval from the Siriraj Institutional Review Board (Si‐137/2022) in Bangkok, Thailand. Additionally, each participating UCARE site conducted a local review and, if needed, obtained approval for the study from its respective Institutional Review Board. PREVALENCE‐D was conducted via the Internet and did not collect personal identifying information.

### 2.2 Sample Size Calculation

The participating UCAREs were instructed to recruit at least 3000 individuals on the basis of an estimated SD prevalence of 2% with a 95% confidence interval (CI) of 2% ± 0.5%. Each center aimed to enroll at least 1000 participants per age group: the student group (18–24 years), the working‐age group (25–60 years), and older people (> 60 years, according to the World Health Organization [21]).

### 2.3 Development, Structure, and Translation of the PREVALENCE‐D Questionnaire

The principal investigator (K.K.) and her team created the PREVALENCE‐D questionnaire in English. This version was reviewed, edited, and approved by the steering committee members, all of whom were UCARE members and global experts in urticaria.

The introduction of the questionnaire explained the study aims and described dermographism using plain language and illustrative images. For instance, an image of a red, flat line on human skin depicted PD, whereas an image of a red, raised line indicated urticarial dermographism. The absence or presence of itch distinguished SimUD from SD, respectively. As SD is chronic in nature, in order for participants to distinguish chronic, recurrent, or ongoing itchy urticarial dermographism from transient episodes (last only a few days) of itchy lesions, the term “chronic SD” was used in the questionnaire. At the end of the introduction, prospective participants were asked to indicate their willingness to be involved in the study and to answer the PREVALENCE‐D questionnaire (Figure S1). Google Forms was used for data collection, and the participating UCAREs translated the English questionnaire directly within the platform into their respective local languages.

### 2.4 Guidance for Distributing the PREVALENCE‐D Questionnaire

To recruit a demographically representative subject group that reflects the general population, the participating UCAREs received guidance to distribute an internet‐based questionnaire. Each center was advised to use age‐appropriate platforms tailored to each age group, such as websites or other public social media platforms for university students, workers, and the elderly. Health‐related websites or social media platforms associated with skin diseases, and a dominant gender representation (e.g., military‐related or women's club) were excluded.

### 2.5 Data Gathering and Analysis

Each site converted its Google Forms responses into an Excel file. For open‐ended responses, local languages were translated into English before submission to the principal investigator for statistical analysis. Sex and age data were collected through self‐reporting by the participants. Because the distributions of sex and age in each sample might not match those of the general population, sex‐ and age‐adjusted prevalence estimates were computed based on each country's population (Table S2) with 95% CIs (Figure S2). The data were categorized by sex, age group, and country.

Statistical comparisons of SD prevalence by sex and age group were performed via Pearson's chi‐squared test or Fisher's exact test, with Bonferroni correction for multiple comparisons. A *p* value < 0.05 was considered statistically significant. Analyses were conducted using IBM SPSS Statistics, version 30.0 (IBM Corp, Armonk, NY, USA) and PASS, version 21.0 (NCSS LLC, Kaysville, UT, USA). Graphs were generated via GraphPad Prism, version 10.0 (GraphPad Software, Boston, MA, USA).

### 2.6 Prevalence Determination

The prevalence rates for each type of dermographism were derived from participants' responses to the questionnaire (Figure S1). The point prevalence of PD was defined as the proportion of participants reporting transient erythema without wheals or itch at the time of the survey. The point prevalence of SimUD was the proportion of participants reporting urticarial dermographism without itch, whereas the SD point prevalence was the proportion reporting recurrent urticarial dermographism with itch at the time of the questionnaire. Lifetime prevalence rates for PD, SimUD, and SD were defined as the proportion of participants who had ever experienced each subtype.

## 3 Results

**FIGURE 2:**
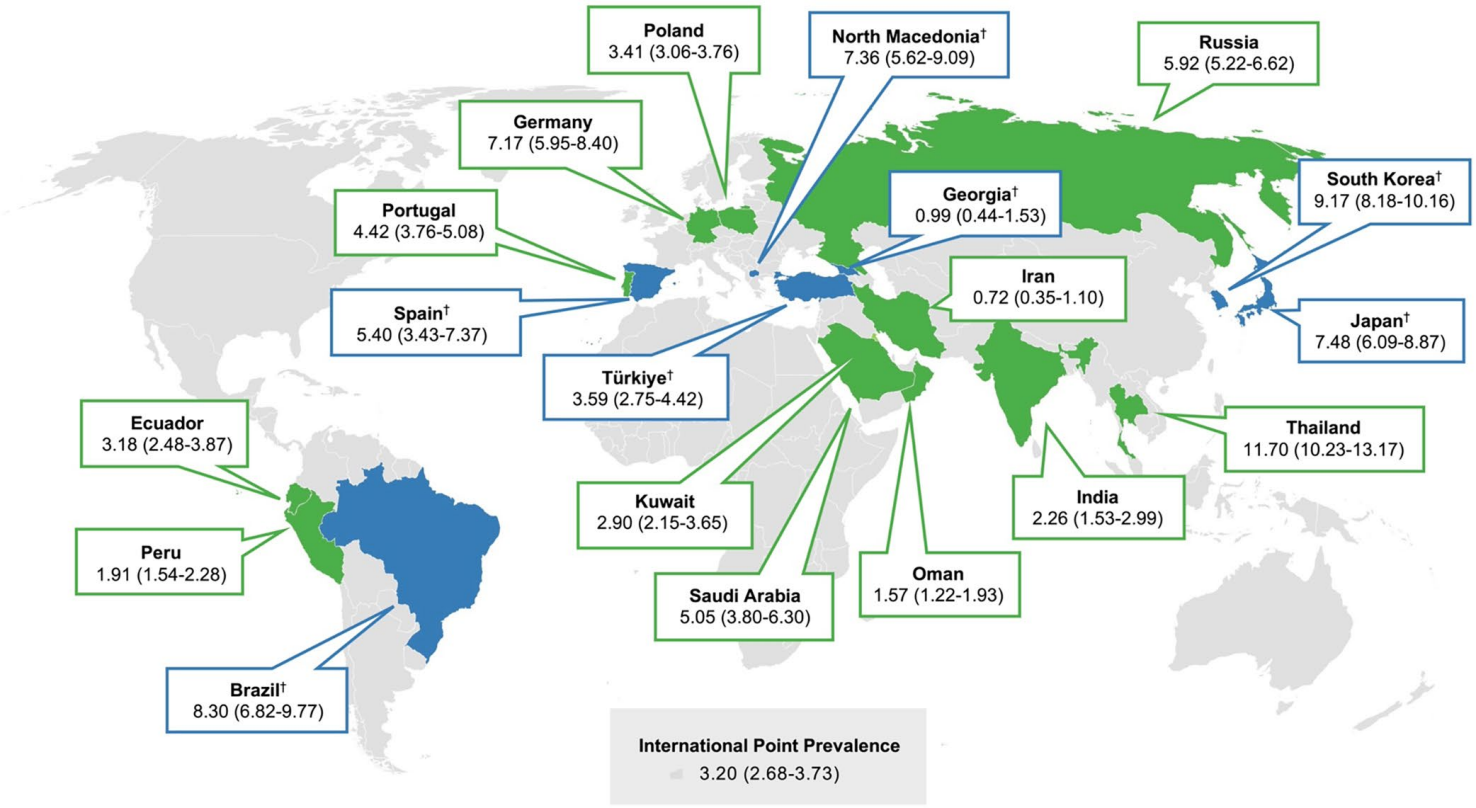
Sex‐ and age‐adjusted point prevalence of each type of dermographism. Sex‐ and age‐adjusted point prevalence of symptomatic dermographism was presented as percentages with 95% confidence intervals across countries. Point prevalence estimates were adjusted for sex and age distribution based on each country's population. Standard error of the sex‐ and age‐adjusted prevalence in each country was calculated based on the principle of stratified random sampling where stratum was defined according to sex, and age group. ^†^The blue‐shaded countries did not reach the target population and were not included in the international prevalence analysis. [Correction added on 18 September 2025, after first online publication: Figure 2 has been replaced.]

Of the 19 countries participating in PREVALENCE‐D, 12 provided complete data for each age group: Ecuador, Germany, India, Iran, Kuwait, Oman, Peru, Poland, Portugal, Russia, Saudi Arabia, and Thailand (Figure 2). These datasets were pooled for the international prevalence analysis, with each country's prevalence reported in the Tables S3.1–S3.12. The remaining seven countries—Brazil, Georgia, Japan, North Macedonia, South Korea, Spain, and Türkiye—had fewer than 1000 participants in at least one age group and were analyzed separately (Tables S3.13–S3.19).

Participants from each center were recruited through various channels, with social media being the most common (94.7%) (Table S4). Across the 19 participating countries, non‐participation rates ranged from 0.05% to 9.55%, with a median of 1.28% (Table S5). In total, 59,543 participants were included in this study. Table 1 presents the crude and sex‐ and age‐adjusted international point and lifetime prevalence of each dermographism subtype. Table 2 provides details by country.

**TABLE 1:** Prevalence of each type of dermographism among all participants internationally (*n* = 59,543).

Age group	Population aged ≥ 18 years of 12 countries in 2023^b^, *N* (%)	Sample in this study, *n* (%)	Point prevalence						Lifetime prevalence					
			Physiological red dermographism		Simple urticarial dermographism		Symptomatic dermographism		Physiological red dermographism		Simple urticarial dermographism		Symptomatic dermographism	
			*n*/*N*	% (95% CI)	*n*/*N*	% (95% CI)	*n*/*N*	% (95% CI)	*n*/*N*	% (95% CI)	*n*/*N*	% (95% CI)	*n*/*N*	% (95% CI)
**Male**														
18–24 years	118,124,000 (8.3)	6656 (11.2)	1837/6656	27.60 (26.35–28.89)	195/6656	2.93 (2.53–3.37)	168/6656	2.52 (2.16–2.94)	3078/6656	46.24 (44.62–47.91)	754/6656	11.33 (10.53–12.17)	445/6656	6.69 (6.08–7.34)
25–60 years	490,909,000 (34.6)	9033 (15.2)	1993/9033	22.06 (21.11–23.05)	176/9033	1.95 (1.67–2.26)	274/9033	3.03 (2.69–3.42)	3873/9033	42.88 (41.54–44.25)	854/9033	9.45 (8.83–10.11)	725/9033	8.03 (7.45–8.63)
> 60 years	110,489,000 (7.7)	7785 (13.1)	1071/7785	13.76 (12.95–14.61)	97/7785	1.25 (1.01–1.52)	162/7785	2.08 (1.77–2.43)	2453/7785	31.51 (30.27–32.78)	546/7785	7.01 (6.44–7.63)	514/7785	6.60 (6.04–7.20)
% Crude prevalence (95% CI)			4901/23,474	20.88 (20.30–21.47)	468/23,474	1.99 (1.82–2.18)	604/23,474	2.57 (2.37–2.79)	9404/23,474	40.06 (39.26–40.88)	2154/23,474	9.18 (8.79–9.57)	1684/23,474	7.17 (6.84–7.53)
% Age‐adjusted prevalence (95% CI)				8.71 (7.85–9.57)		0.97 (0.78–1.16)		3.02 (2.16–3.89)		34.41 (31.95–36.87)		4.65 (4.03–5.27)		5.79 (4.77–6.81)
**Female**														
18–24 years	107,849,000 (7.6)	11,484 (19.3)	4012/11,484	34.94 (33.86–36.03)	533/11,484	4.64 (4.26–5.05)	404/11,484	3.52 (3.18–3.88)	6457/11,484	56.23 (54.86–57.61)	2060/11,484	17.94 (17.17–18.73)	921/11,484	8.02 (7.51–8.56)
25–60 years	461,407,000 (32.5)	15,310 (25.7)	4421/15,310	28.88 (28.03–29.74)	597/15,310	3.90 (3.59–4.23)	914/15,310	5.97 (5.59–6.37)	8031/15,310	52.46 (51.31–53.62)	2161/15,310	14.11 (13.53–14.72)	1753/15,310	11.45 (10.92–12.00)
> 60 years	130,875,000 (9.2)	9275 (15.6)	1887/9275	20.35 (19.44–21.28)	193/9275	2.08 (1.80–2.40)	294/9275	3.17 (2.82–3.55)	3671/9275	39.58 (38.31–40.88)	810/9275	8.73 (8.14–9.36)	729/9275	7.86 (7.30–8.45)
% Crude prevalence (95% CI)			10,320/36,069	28.61 (28.06–29.17)	1323/36,069	3.67 (3.47–3.87)	1612/36,069	4.47 (4.25–4.69)	18,159/36,069	50.35 (49.62–51.08)	5031/36,069	13.95 (13.57–14.34)	3403/36,069	9.43 (9.12–9.76)
% Age‐adjusted prevalence (95% CI)				11.36 (10.74–11.98)		1.46 (1.27–1.65)		3.39 (2.80–3.97)		32.51 (30.95–34.07)		6.16 (5.66–6.66)		6.09 (5.41–6.78)
Total	1,419,653,000 (100.0)	59,543 (100.0)	15,221/59,543	25.56 (25.16–25.97)	1791/59,543	3.01 (2.87–3.15)	2216/59,543	3.72 (3.57–3.88)	27,563/59,543	46.29 (45.75–46.84)	7185/59,543	12.07 (11.79–12.35)	5087/59,543	8.54 (8.31–8.78)
% Crude prevalence (95% CI)				25.56 (25.16–25.97)		3.01 (2.87–3.15)		3.72 (3.57–3.88)		46.29 (45.75–46.84)		12.07 (11.79–12.35)		8.54 (8.31–8.78)
% Sex‐ and age‐adjusted prevalence^a^ (95% CI)				10.02 (9.48–10.55)		1.21 (1.08–1.35)		3.20 (2.68–3.73)		33.47 (32.01–34.94)		5.39 (4.99–5.79)		5.94 (5.32–6.56)

*Note:* From 12 countries (i.e., Ecuador, Germany, India, Iran, Kuwait, Oman, Peru, Poland, Portugal, Russia, Saudi Arabia, and Thailand), the international point and lifetime prevalence of each type of dermographism (i.e., physiological dermographism, simple urticarial dermographism, and symptomatic dermographism) was reported as crude rates, separately by sex and age group. Additionally, for males and females, the age‐adjusted prevalence was reported along with the 95% confidence interval.

^a^ Prevalence is adjusted based on 72 strata by 12 countries, 2 sexes, and 3 age groups.

^b^ Standard population was obtained from United Nations, World Population Prospects 2024.

**TABLE 2:** Sex‐ and age‐adjusted prevalence of each type of dermographism across 12 countries (*n* = 59,543).

	Sample size (*n*)	Physiological red dermographism		Simple urticarial dermographism		Symptomatic dermographism	
		PP^a^, % (95% CI)	LP^a^, % (95% CI)	PP^a^, % (95% CI)	LP^a^, % (95% CI)	PP^a^, % (95% CI)	LP^a^, % (95% CI)
International	59,543	10.02 (9.48–10.55)	33.47 (32.01–34.94)	1.21 (1.08–1.35)	5.39 (4.99–5.79)	3.20 (2.68–3.73)	5.94 (5.32–6.56)
Ecuador	3582	13.81 (12.49–15.14)	57.34 (55.36–59.32)	3.38 (2.70–4.05)	24.20 (22.50–25.90)	3.18 (2.48–3.87)	10.76 (9.54–11.99)
Germany	3541	19.33 (17.49–21.17)	56.31 (54.16–58.47)	1.95 (1.36–2.54)	16.54 (14.95–18.13)	7.17 (5.95–8.40)	11.06 (9.62–12.50)
India	3092	3.92 (3.21–4.63)	26.87 (24.23–28.34)	0.47 (0.17–0.77)	1.94 (1.38–2.50)	2.26 (1.53–2.99)	3.13 (2.29–3.98)
Iran	3253	4.65 (3.73–5.57)	21.47 (19.66–23.28)	1.95 (1.36–2.55)	5.98 (4.97–6.98)	0.72 (0.35–1.10)	2.15 (1.50–2.80)
Kuwait	3556	6.81 (5.64–7.98)	21.23 (19.32–23.10)	2.00 (1.41–2.59)	9.27 (7.98–10.55)	2.90 (2.15–3.65)	6.05 (4.97–7.13)
Oman	7106	57.90 (56.27–59.53)	61.57 (59.96–63.18)	1.06 (0.75–1.34)	4.14 (3.51–4.77)	1.57 (1.22–1.93)	3.31 (2.75–3.87)
Peru	6447	8.65 (7.81–9.48)	40.78 (39.37–42.19)	2.65 (2.17–3.13)	15.22 (14.19–16.24)	1.91 (1.54–2.28)	8.08 (7.32–8.84)
Poland	12,084	29.39 (28.49–30.28)	38.93 (37.96–39.89)	2.89 (2.57–3.20)	6.43 (5.97–6.88)	3.41 (3.06–3.76)	6.07 (5.60–6.53)
Portugal	4254	12.80 (11.72–13.88)	48.73 (47.05–50.40)	2.15 (1.71–2.60)	16.73 (15.51–17.94)	4.42 (3.76–5.08)	10.91 (9.89–11.93)
Russia	6335	40.76 (39.35–42.17)	66.61 (65.18–68.04)	5.83 (5.14–6.52)	18.67 (17.49–19.85)	5.92 (5.22–6.62)	17.34 (16.18–18.51)
Saudi Arabia	3046	9.95 (8.26–11.65)	17.34 (15.23–19.44)	0.36 (0.06–0.67)	1.92 (1.15–2.70)	5.05 (3.80–6.30)	7.17 (5.73–8.61)
Thailand	3247	34.73 (32.66–36.80)	69.61 (67.56–71.67)	5.29 (4.35–6.23)	17.55 (15.83–19.28)	11.70 (10.23–13.17)	26.67 (24.65–28.70)

*Note:* For each of the 12 countries, the sex‐ and age‐adjusted point and lifetime prevalence of each type of dermographism was reported. Adjustments were based on the sex and age distribution of the population in each country. The standard error of the adjusted rate was computed based on the principles of stratified random sampling.

Abbreviations: LP, lifetime prevalence; PP, point prevalence.

^a^ Point and lifetime prevalence are adjusted based on sex and age group distribution in each country's population.

For SD, the international sex‐ and age‐adjusted point prevalence was 3.20% (95% CI 2.68%–3.73%), ranging from 0.72% (95% CI 0.35%–1.10%) in Iran to 11.70% (95% CI 10.23%–13.17%) in Thailand. The corresponding international lifetime prevalence was 5.94% (95% CI 5.32%–6.56%), varying from 2.15% (95% CI 1.50%–2.80%) in Iran to 26.67% (95% CI 24.65%–28.70%) in Thailand.

The international sex‐ and age‐adjusted point prevalence of PD was 10.02% (95% CI 9.48%–10.55%), ranging from 3.92% (95% CI 3.21%–4.63%) in India to 57.90% (95% CI 56.27%–59.53%) in Oman. Its international lifetime prevalence was 33.47% (95% CI 32.01%–34.94%). This measure ranged from 17.34% (95% CI 15.23%–19.44%) in Saudi Arabia to 69.61% (95% CI 67.56%–71.67%) in Thailand.

For SimUD, the international sex‐ and age‐adjusted point prevalence was 1.21% (95% CI 1.08%–1.35%), varying from 0.36% (95% CI 0.06%–0.67%) in Saudi Arabia to 5.83% (95% CI 5.14%–6.52%) in Russia. The international lifetime prevalence of SimUD was 5.39% (95% CI 4.99%–5.79%), with estimates ranging from 1.92% (95% CI 1.15%–2.70%) in Saudi Arabia to 24.20% (95% CI 22.50%–25.90%) in Ecuador.

Of 12,421 participants with a history of PD, 371 (3.0%) had current SD and of 5413 participants with a history of SimUD, 28 (0.5%) currently had SD. Among the three age groups, the working‐age group (25–60 years) presented the highest international point and lifetime prevalence of SD, with 3.80% (95% CI 3.04%–4.57%) and 6.70% (95% CI 5.81%–7.59%), respectively. Overall, the international prevalence was higher in females. In particular, the international female age‐adjusted prevalence of SD reached 3.39% (95% CI 2.80%–3.97%) for point prevalence and 6.09% (95% CI 5.41%–6.78%) for lifetime prevalence (Table 1).

**FIGURE 3:**
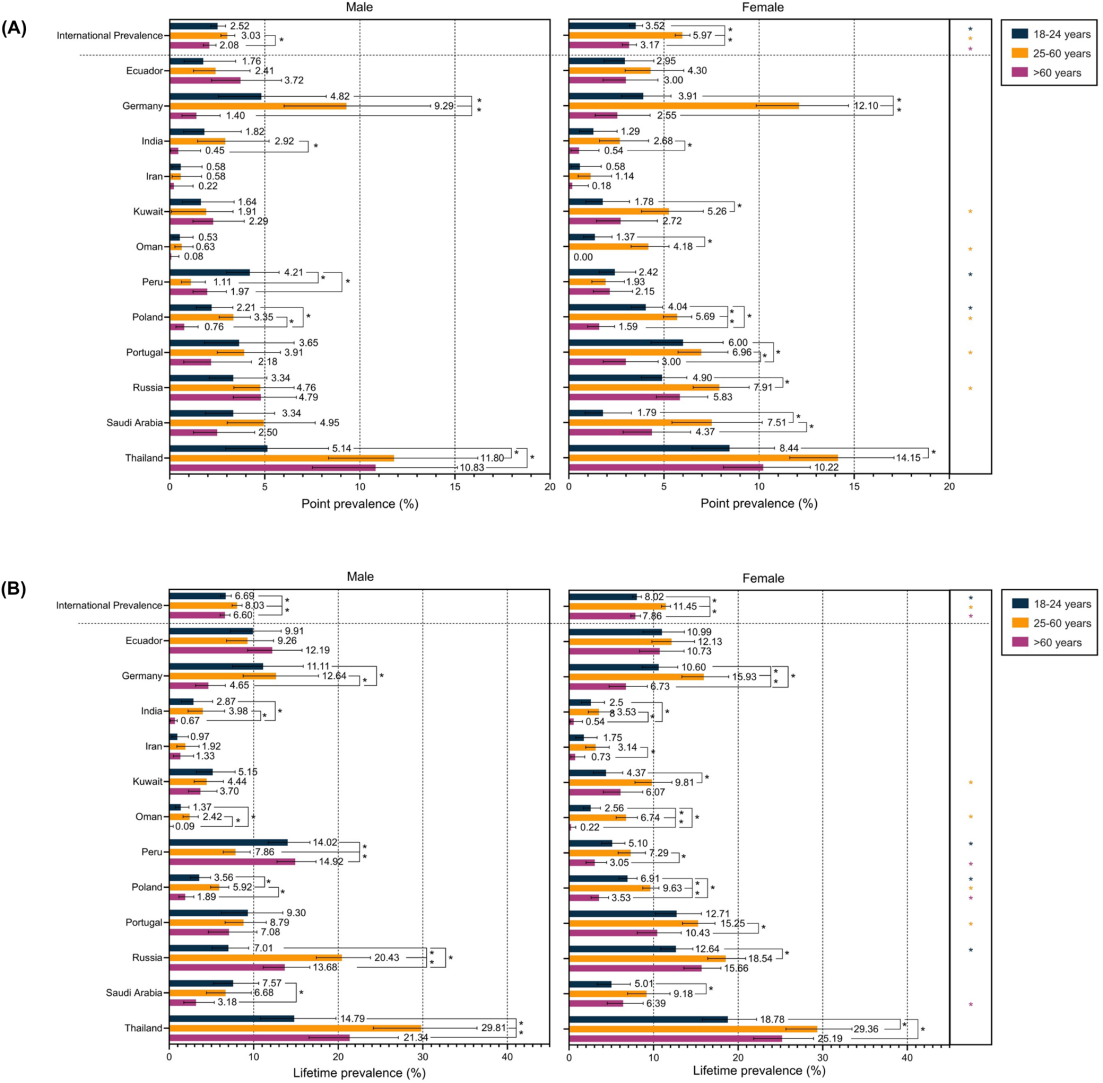
Comparison of the point and lifetime prevalence of symptomatic dermographism by sex and age group. (A and B) Point and lifetime prevalence of symptomatic dermographism are presented as percentages with 95% confidence intervals, stratified by sex and age group across 12 countries. Comparative analysis of symptomatic dermographism prevalence across age groups and between males and females was performed using two‐tailed Pearson's chi‐squared test or Fisher's exact test, with Bonferroni correction for multiple comparisons. A *p* value < 0.05 was considered statistically significant. Standard error of the adjusted rated was calculated based on the principle of stratified random sampling. (A) Comparison of the point prevalence of symptomatic dermographism by sex and age group. (B) Comparison of the lifetime prevalence of symptomatic dermographism by sex and age group. *p value < 0.05 between age groups was considered statistically significant. Green, orange, purple * for *p* value between sexes in age group 18–24, 25–60, > 60 years, respectively.

Figure 3 illustrates the point and lifetime prevalence of SD by age group for males and females. The detailed data are presented in Tables S6 and S7. In males, the point prevalence of SD was highest in the 25–60 years age group and differed significantly from that in the over 60 years age group. In females, the 25–60 years age group also had the highest point prevalence, with significant differences between this group and all other age groups. In terms of lifetime prevalence, significant differences emerged in both sexes between the 25 and 60 age group and all other age groups. The highest prevalence occurred in the 25–60 age group, with generally higher rates in females than in males.

## 4 Discussion

The PREVALENCE‐D study is the largest cross‐sectional international investigation of dermographism in adults. It comprehensively analyzes the international prevalence of PD, SimUD, and SD. The questionnaire was developed by world urticaria experts, who incorporated plain language and illustrative images to ensure accurate participant self‐diagnosis of each dermographism subtype. The data analyses were adjusted for sex and age to provide estimates that are applicable to the general population.

Although a sample size of 3000 respondents per country may not fully capture the prevalence of dermographism in nations with millions of inhabitants, representativeness can be approximated by comparing the sex and age distributions of respondents (≥ 18 years) with national data. The 95% CI of each country's prevalence helps quantify precision, and pooling all participating countries' data narrows this interval further, yielding a more robust international estimate of dermographism.

For SD, the international sex‐ and age‐adjusted point prevalence was 3.20%, whereas the international lifetime prevalence was 5.94%. PD had a point prevalence of 10.02% and a lifetime prevalence of 33.47%. SimUD showed a point prevalence of 1.21% and a lifetime prevalence of 5.39%. A minority of participants with ongoing SD had a history of PD (≤ 3%) or SimUD (< 1%).

Our findings for SD align with earlier reports of a prevalence of 0.12%–5.00% (Table S1). Kirby et al. [13] remain the only prior study to examine dermographism in a general population (*n* = 2813), although that investigation did not address pruritus. Moreover, previous studies offered no prevalence estimates for PD because PD lacked a clear universal definition. The large sample size of PREVALENCE‐D and well‐defined cutaneous response subtypes enabled more precise prevalence estimates. The high lifetime prevalence of SD emphasizes appropriate healthcare resource allocation.

A higher prevalence of SD from our study was observed in East Asia (7.48%–11.70%), followed by Europe (3.41%–7.36%) and the Middle East (0.72%–5.05%). A recent meta‐analysis reported a higher point prevalence of chronic urticaria in Asia than in Europe and North America [22]. Various factors, including ethnicity, geographic region, air pollution, and weather conditions, have been reported to contribute to the prevalence of urticaria [15, 22] and dermographism [23]. Muizzuddin et al. reported a significantly weaker barrier strength and a lower maturation index, which is a measure of stratum corneum protein crosslinking, in East Asians compared to Caucasians [24]. Additionally, Asian skin has been shown to be more sensitive and reactive compared to Caucasian skin [25]. These findings may explain the higher observed rate of SD in East Asians, as increased skin sensitivity could render their skin more reactive to triggering factors such as friction or scratching. Thus, our finding supports that ethnicity and geographic location may contribute to the observed differences in prevalence among countries. Further investigation is warranted to better understand these associations.

The international point and lifetime age‐adjusted prevalence rates of SD were greater in females (3.39% and 6.09%, respectively) than in males (3.02% and 5.79%, respectively). Kirby et al. [13] similarly reported a female predominance. Other studies have found that 55.90%–75.20% of patients with SD are female [5, 26, 27, 28]. This reflects the gender distribution pattern observed in chronic spontaneous urticaria (CSU) [29], which occurs at least twice as often in females as in males. By age group, the working‐age category (25–60 years) had the highest prevalence of SD, at 3.80% for point prevalence and 6.70% for lifetime prevalence.

The associations between CSU and autoimmune diseases are well established. Studies have reported that immunoglobulin G (IgG) autoantibodies to IgE or the IgE receptor (FcεRI), as well as IgE antibodies against autoantigens, result in mast cell and basophil activation and subsequent urticarial symptoms [30]. However, the pathophysiology of SD is not yet fully understood. Neo‐allergens might be produced in the skin after scratching followed by the development of IgE autoantibodies, IgE‐mediated mast cell activation, the release of vasoactive mediators, and wheal formation [25]. The successful treatment of SD with omalizumab, an anti‐IgE monoclonal antibody [3, 26], further supports an IgE‐dependent mechanism. Patients with SD exhibit higher total serum IgE levels than those without dermographism [31]. These findings suggest that SD may share pathophysiological features with CSU. These features include a female predominance and a higher prevalence in middle‐aged individuals, both of which align with an underlying autoimmune process [32]. Additional research is needed to clarify the pathophysiological mechanisms that might explain our findings.

This study has some limitations. First, only participants aged 18 years or older were included, which restricts generalization to younger age groups. Second, because the questionnaire was distributed online through an open platform, it was not possible to calculate a response rate. Numerous individuals might have received and reviewed it without providing a response. Among the 19 participating countries, the proportion of individuals who declined to participate after reviewing the survey introduction ranged from 0.05% to 9.55%, with a median non‐participation rate of 1.28%. A non‐participation rate below 15% in each country is considered acceptable according to established response rate standards [33]. These findings may indicate a broad willingness among participants to engage with the study across diverse geographic and cultural contexts, rather than a response limited to individuals with a pre‐existing interest in skin conditions. Third, participants who were not able to access public social media or online platforms could not participate in this study. Fourth, the questionnaire‐based design relied on self‐reported data, introducing potential recall bias and subjective question understanding and reporting of the disease and symptoms. Despite the provision of detailed explanations and illustrative images to help participants distinguish between cutaneous response subtypes, some inconsistencies in self‐assessment may nevertheless have occurred. Moreover, skin tone data (e.g., Fitzpatrick type) were not collected, which may affect interpretation of erythema and skin response variability across diverse populations.

However, individuals themselves are often able to distinguish redness even in very dark skin, although it becomes more difficult for external investigators as skin tone darkens. Fifth, despite our efforts to reach the general population, individuals with SD symptoms, even though mild, may have been more motivated to participate, possibly leading to a selection bias and an overestimation of prevalence. Lastly, although this study included participants from 19 countries across multiple continents, no countries from the African continent participated in the study. This geographic gap, especially the underrepresentation of populations with darker skin phototypes, restricts the generalizability of our prevalence estimates. Future studies should aim to incorporate data from underrepresented geographic regions to enhance the comprehensiveness and global representativeness of dermographism prevalence estimates.

## 5 Conclusion

The PREVALENCE‐D study provides an extensive overview of the prevalence of SD in adults worldwide, with an international sex‐ and age‐adjusted point prevalence of 3.20% and a lifetime prevalence of 5.94%. A higher prevalence was observed among females and in the working‐age population, which can be very important, especially in professions where manual work is required. These findings emphasize the need for public health planning, healthcare policy development, targeted clinical trials, and trend monitoring in follow‐up studies.

## Author Contributions

K.K., Ma.M., A.M.G.‐A., J.A.B., P.R.C., K.G., Y.‐M.Y., M.A., and Me.M. were responsible for this study design and developed the research strategy. K.K., Ma.M., N.J., Y.N., and W.P. created a prototype Google Forms for data collection and managed the distribution of questionnaires to the participating countries. K.K., Ph.S., J.L., and T.W. performed the data extraction. K.K., Ma.M., Pa.S., C.K., Ph.S., J.L., and T.W. performed the formal analyses. K.K., Ma.M., T.Z., L.C., and C.K. prepared the visualization. K.K., Ma.M., T.Z., J.A.B., M.R., Pa.S., P.K., C.K., L.C., Ph.S., J.L., T.W., and T.P. wrote the first draft of the manuscript. All investigators contributed to the study recruitment, data acquisition, manuscript editing, and review of the final submitted version. All authors were not precluded from accessing data in the study, and they accepted responsibility to submit for publication.

## Conflicts of Interest

K.K. received educational speaker fees from Novartis, Menarini, Sanofi Genzyme, and Takeda, outside of submitted work. J.A.B. is or recently was a principal investigator and consultant of Sanofi‐Regeneron, Novartis, Genentech, Teva, AZ, Amgen, Celldex, Jasper, Allakos, Blueprint Medicine, Escient/Incyte. M.A. is or recently was a speaker and advisory board honoraria from GSK, Sanofi, Novartis, and AstraZeneca, outside of submitted work. N.A.A. is or recently was a speaker honoraria from Novartis. A.A. was a speaker for Novartis, outside of submitted work. L.F.E. received fees for consultancy from Sanofi, travel grants from Sanofi, and as a speaker from Novartis, Sanofi, and Celtrion. R.F.J.C. received fees for lectures or consultings out of this article from the following laboratories: Pfizer, Sanofi, Novartis, Lilly. A.M.G.‐A. is or recently was a speaker and/or advisor for and/or has received research funding from Almirall, Amgen, AstraZeneca, Avene, Blue‐Print, Celldex, Escient Pharmaceuticals, Genentech, GSK, Harmonic Bio, Instituto Carlos III‐ FEDER, Jaspers, Leo Pharma, Menarini, Mitsubishi Tanabe Pharma, Noucor, Novartis, Sanofi‐Regeneron, Septerna, Servier, Thermo Fisher Scientific, Uriach Pharma. N.I. is or recently was a speaker and/or has received research funding from GSK, Tanabe‐Mitsubishi Pharma, Novartis, Sanofi‐Regeneron, Taiho, Torii, and Kyowa‐Kirin. M.K. was a speaker and/or advisor for and/or has received research funding from Abidi Pharma, Alhavi Pharma, AstraZeneca, Actoverco, Ofogh Tolid Darou pars, Kimia salamat nikan, CinnaGen, Sanofi, GlaxoSmithKline, and Danon, outside of submitted work. P.K. was a speaker and/or consultant for BioCryst, Merus, Novartis, and ValenzaBio, outside of submitted work. Me.M. is or recently was a speaker, advisor, and/or received research funding from Jasper Therapeutics, Celltrion, Celldex, Takeda, BioCryst, Annexon, and Roche. R.Ö.K. gave a speak for Novartis, AbbVie, Lilly, and La Roche Posay. P.T. received educational speaker fees from Novartis, Menarini, Sanofi Genzyme, outside of submitted work. Ma.M. is or recently was a speaker and/or advisor for and/or has received research funding from Allakos, Alexion, Alvotech, Almirall, Amgen, Aquestive, argenx, AstraZeneca, Celldex, Celltrion, Clinuvel, Escient, Evommune, Excellergy, GSK, Incyte, Jasper, Kashiv, Kyowa Kirin, Leo Pharma, Lilly, Menarini, Mitsubishi Tanabe Pharma, Moxie, Noucor, Novartis, Orion Biotechnology, Resonance Medicine, Sanofi/Regeneron, Santa Ana Bio, Septerna, Servier, Third Harmonic Bio, ValenzaBio, Vitalli Bio, Yuhan Corporation, and Zurabio. T.Z. has received institutional funding for research and/or honoraria for lectures and/or consulting from Amgen, AstraZeneca, AbbVie, ALK, Almirall, Astellas, Bayer Health Care, Bencard, Berlin Chemie, Blueprint Medicines, FAES, HAL, Henkel, Kryolan, Leti, L'Oreal, Meda, Menarini, Merck, MSD, Novartis, Pfizer, Sanofi, Stallergenes, Takeda, Teva, UCB, and Uriach; in addition, he is a member of Allergic Rhinitis and its Impact on Asthma (ARIA), the German Society for Allergy and Clinical Immunology (DGAKI), the European Centre for Allergy Research Foundation (ECARF), the Global Allergy and Asthma European Network (GA^2^LEN) and World Allergy Organization (WAO). The other authors declare no conflicts of interest.

## Supporting information


**Figure S1:** The flow diagram of questions in the questionnaire.
**Figure S2:** Supplementary methods for calculation of international sex‐ and age‐ adjusted.
**Table S1:** Studies that mentioned the prevalence of dermographism.
**Table S2:** Total population by age group and sex among each participating country in 2023.
**Table S3.1:** Prevalence of each type of dermographism of all participants in Ecuador.
**Table S3.2:** Prevalence of each type of dermographism of all participants in Germany.
**Table S3.3:** Prevalence of each type of dermographism of all participants in India.
**Table S3.4:** Prevalence of each type of dermographism of all participants in Iran.
**Table S3.5:** Prevalence of each type of dermographism of all participants in Kuwait.
**Table S3.6:** Prevalence of each type of dermographism of all participants in Oman.
**Table S3.7:** Prevalence of each type of dermographism of all participants in Peru.
**Table S3.8:** Prevalence of each type of dermographism of all participants in Poland.
**Table S3.9:** Prevalence of each type of dermographism of all participants in Portugal.
**Table S3.10:** Prevalence of each type of dermographism of all participants in Russia.
**Table S3.11:** Prevalence of each type of dermographism of all participants in Saudi Arabia.
**Table S3.12:** Prevalence of each type of dermographism of all participants in Thailand.
**Table S3.13:** Prevalence of each type of dermographism of all participants in Brazil.
**Table S3.14:** Prevalence of each type of dermographism of all participants in Georgia.
**Table S3.15:** Prevalence of each type of dermographism of all participants in Japan.
**Table S3.16:** Prevalence of each type of dermographism of all participants in North Macedonia.
**Table S3.17:** Prevalence of each type of dermographism of all participants in South Korea.
**Table S3.18:** Prevalence of each type of dermographism of all participants in Spain.
**Table S3.19:** Prevalence of each type of dermographism of all participants in Türkiye.
**Table S4:** Participant recruitment: channels to distribute the internet‐based questionnaire.
**Table S5:** Number of participating responses.
**Table S6:** Comparison of prevalence of symptomatic dermographism by age group across 12 countries 26.
**Table S7:** Comparison of prevalence of symptomatic dermographism by sex across 12 countries.

## Data Availability

The data that support the findings of this study are available from the corresponding author upon reasonable request.
