# Supplementary material for: The Prevalence of Symptomatic Dermographism: Results of the International UCARE PREVALENCE‐D Study
Source: Allergy. 2025 Sep 13;81(2):468–79. doi: 10.1111/all.70047 (PMC12862545; doi:10.1111/all.70047)
Supplement: Supplementary file 1 — Figure S1: The flow diagram of questions in the questionnaire. Figure S2: Supplementary methods for calculation of international sex‐ and age‐ adjusted. Table S1: Studies that mentioned the prevalence of dermographism. Table S2: Total population by age group and sex among each participating country in 2023. Table S3.1: Prevalence of each type of dermographism of all participants in Ecuador. Table S3.2: Prevalence of each type of dermographism of all participants in Germany. Table S3.3: Prevalence of each type of dermographism of all participants in India. Table S3.4: Prevalence of each type of dermographism of all participants in Iran. Table S3.5: Prevalence of each type of dermographism of all participants in Kuwait. Table S3.6: Prevalence of each type of dermographism of all participants in Oman. Table S3.7: Prevalence of each type of dermographism of all participants in Peru. Table S3.8: Prevalence of each type of dermographism of all participants in Poland. Table S3.9: Prevalence of each type of dermographism of all participants in Portugal. Table S3.10: Prevalence of each type of dermographism of all participants in Russia. Table S3.11: Prevalence of each type of dermographism of all participants in Saudi Arabia. Table S3.12: Prevalence of each type of dermographism of all participants in Thailand. Table S3.13: Prevalence of each type of dermographism of all participants in Brazil. Table S3.14: Prevalence of each type of dermographism of all participants in Georgia. Table S3.15: Prevalence of each type of dermographism of all participants in Japan. Table S3.16: Prevalence of each type of dermographism of all participants in North Macedonia. Table S3.17: Prevalence of each type of dermographism of all participants in South Korea. Table S3.18: Prevalence of each type of dermographism of all participants in Spain. Table S3.19: Prevalence of each type of dermographism of all participants in Türkiye. Table S4: Participant recruitment: channels to distribute the inte [file ALL-81-468-s001.pdf]

The International Prevalence of Symptomatic Dermographism: Results of the UCARE PREVALENCE-D Study

SUPPORTING INFORMATION

TABLE OF CONTENTS

SUPPLEMENTARY TABLES AND FIGURES 2

Supplementary Table S1. Studies that mentioned the prevalence of dermographism 2

Supplementary Table S2. Total population by age group and sex among each participating country in 2023 4

Supplementary Table S3.1. Prevalence of each type of dermographism of all participants in Ecuador 5

Supplementary Table S3.2. Prevalence of each type of dermographism of all participants in Germany 6

Supplementary Table S3.3. Prevalence of each type of dermographism of all participants in India 7

Supplementary Table S3.4. Prevalence of each type of dermographism of all participants in Iran 8

Supplementary Table S3.5. Prevalence of each type of dermographism of all participants in Kuwait 9

Supplementary Table S3.6. Prevalence of each type of dermographism of all participants in Oman 10

Supplementary Table S3.7. Prevalence of each type of dermographism of all participants in Peru 11

Supplementary Table S3.8. Prevalence of each type of dermographism of all participants in Poland 12

Supplementary Table S3.9. Prevalence of each type of dermographism of all participants in Portugal 13

Supplementary Table S3.10. Prevalence of each type of dermographism of all participants in Russia 14

Supplementary Table S3.11. Prevalence of each type of dermographism of all participants in Saudi Arabia 15

Supplementary Table S3.12. Prevalence of each type of dermographism of all participants in Thailand 16

Supplementary Table S3.13. Prevalence of each type of dermographism of all participants in Brazil 17

Supplementary Table S3.14. Prevalence of each type of dermographism of all participants in Georgia 18

Supplementary Table S3.15. Prevalence of each type of dermographism of all participants in Japan 19

Supplementary Table S3.16. Prevalence of each type of dermographism of all participants in North Macedonia 20

Supplementary Table S3.17. Prevalence of each type of dermographism of all participants in South Korea 21

Supplementary Table S3.18. Prevalence of each type of dermographism of all participants in Spain 22

Supplementary Table S3.19. Prevalence of each type of dermographism of all participants in Türkiye 23

Supplementary Table S4. Participant recruitment: channels to distribute the internet-based questionnaire 24

Supplementary Table S5. Number of participating responses 25

Supplementary Table S6. Comparison of prevalence of symptomatic dermographism by age group across 12 countries 26

Supplementary Table S7. Comparison of prevalence of symptomatic dermographism by sex across 12 countries 28

Supplementary Figure S1. The flow diagram of questions in the questionnaire 31

Supplementary Figure S2. Supplementary methods for calculation of international sex- and age- adjusted prevalence rate 33

REFERENCE 34

Supplementary Table S1. Studies that mentioned the prevalence of dermographism

Author	Country	Study design	Study details	Prevalence	Remarked
Cotton TF. et al[1] *(1917)*	England	Cohort	**Objective:** To investigate prevalence of dermographism<br>**Subjects**: 84 young soldiers who were in the hospital for the condition of "irritable heart"<br>**Testing**: Stroked by a blunt point on the skin in interscapular area	5%	- Not tested in general population
Lewis T. et al [2] *(1924)*	England	Cohort	**Objective:** To determine the relation of dermographism to the vascular phenomenon	5%	- Cited prevalence from Cotton et al.[1]
Walzer A. et al.[3] *(1928)*	USA	Cohort	**Objective:** To test whether dermographism was physiologic response<br>**Subjects**: 48 patients with skin diseases which did not interfere with a test,13 patients with urticaria (n=8) and generalized pruritus (n=5)<br>**Testing**: Stroked on the back	11/48 (23%)<br>11/13 (84%)	- Randomly selected cases in a dermatologic clinic.<br>- 11/48 (23%) of patients with skin diseases responded with a wheal after multiple strokes.<br>- 11/13 (84%) of patients with urticaria and generalized pruritus responded with a wheal after only a single stroke.<br>- A wheal and flare that occurred among patients in the former group was much less prominent than that of the latter group.
Fisher A. et al.[4] *(1953)*	USA	Cohort	**Objective:** To test whether dermographism was inevitable in subacute or chronic urticaria<br>**Subjects**: 100 dermatologic patients without urticaria,<br>100 patients with subacute or chronic urticaria<br>**Testing:** Stroked by a tongue depressor	5/100 (5%)<br><br>6/100 (6%)	- Not tested in general population<br>- 5/100 (5%) of patients with various dermatoses without urticaria, and 6/100 (6%) of patients with subacute or chronic urticaria, responded with distinct dermographism after stroke.
Ebken RK. et al [5] *(1968)*	USA	Cohort	**Objective:** To study the characteristics and prevalence of dermographism<br>**Subjects:** 200 working subjects, including 68 patients with chronic urticaria<br>**Testing:** Scratch test by drawing the point of instrument along volar surface of forearm with total weights of 200, 300 and 400 g	1.5%	- Not tested in general population
Kirby JD. et al [6] *(1971)*	England	Cohort	**Objective:** To investigate prevalence of dermographism in general population<br>**Subjects**: 2813 patients who were subjects attending department of surgery in a routine way and children being routinely examined.<br>**Testing**: Stroked by a round stylus of 0.45 cm^2 area and a pressure of 4900 g/cm^2	4.24%	- Difficulties in obtaining large enough numbers in the older groups.<br>- Tests were conducted by general practitioners and medical officers.
Margolis CF. et al. [7] *(1981)*	USA	Review	**Objective:** To review symptomatic dermographism	5%	- Cited prevalence from Lewis.[2]
Breathnach SM. et al.[8] *(1982)*	England	Cohort	**Objective:** To review symptomatic dermographism: Natural history, clinical features, laboratory investigations and response to therapy	1.5-5%	- Cited prevalence from Lewis [2], Ebken et al [5], and Kirby et al [6]
Matthews CN. et al.[9] *(1983)*	USA	Case control	**Objective:** To compare the effect of chlorpheniramine, hydroxyzine and diazepam, on the degree of itching and the weal size in patients with dermographism	4.25%	- Cited prevalence from Kirby et al [6]
Wong RC, et al. [10] *(1984)*	USA	Review	**Objective:** To review dermographism	4-5%	- Cited prevalence from Kirby et al [6] and Margolis et al[7]
Kontou-Fill K. et al.[11] *(1997)*	Greece	Review	**Objective:** To update physical urticaria (classification and diagnostic guidelines)	2-5%	- Cited prevalence from Kirby et al [6] and Matthews et al.[9]
Martorell A. et al. [12] *(2000)*	Spain	Cohort	**Objective:** To study the prevalence of dermographism in children<br>**Subjects:** 238 children aged 2-14 years from a health care center<br>**Testing:** Dermatographometer with a pressure of 3,200 g/cm^2	24%	- Only pediatric subjects
Schoepke N. et al.[13] *(2015)*	Germany	Cohort	**Objective:** To understand patients' views about the practical aspects of symptomatic dermographism	2-5%	- Cited prevalence from Breathnach et al. [8] and Kontou-Fill et al.[11]
Sanchez-Borges M. et al.[14] *(2017)*	Venezuela	Review	**Objective:** To review physical urticarias and testing methods	2-5%	- It was mentioned in the paper but not cited a reference.
Seo, J. H. et al.[15] *(2019)*	Korea	Cross-sectional	**Objective:** To investigate the epidemiology of various types of urticaria, including dermographism, in Korea and analyze changes in its annual prevalence over 5 years (2010–2014).	0.12%	- The Health Insurance Review and Assessment database was used.
Gu X. et al.[16] *(2021)*	China	Cohort	**Objective:** To study air pollution and meteorological factors associated with dermographism: a population-based study in college students<br>**Subjects:** 16167 college students from three universities in the different regions of China (Changsha, Xiamen, Hohhot)<br>**Testing:** Scratch test with a clean swab stick on the forearm skin by the trained dermatologists	15.6%	- Only college students with a mean age of 18.3 ± 0.8 years

Li, J. et al[17] *(2022)*	China	Cross-sectional	**Objective:** To determine the prevalence, clinical forms, and risk factors of urticaria, including dermographism, in the Chinese population through a large-scale, nationwide, population-based survey. (questionnaire based survey)<br>**Subjects:** 41,041 participants from 35 cities across 31 provinces in China.<br>**Testing:** Scratch test was performed on the forearm skin using a clean swab stick by trained dermatologists to assess the presence of linear wheals.	0.37%	- The population consisted of adults, adolescents, and children aged over 7 years old.
Liu et al.[18] *(2022)*	China	Cohort	**Objective:** To clarify the epidemic distribution characteristics of symptomatic dermographism<br>**Subjects:** 1715 patients with chronic urticaria	36%	- Not tested in general population<br>- Among 1715 patients who completed questionnaire, 615 patients (36%) had symptomatic dermographism.

Supplementary Table S2. Total population by age group and sex among each participating country in 2023 (according to the United Nations[19])

Country	Total Population > 18 years (thousands)	Age group in male N (thousands), (%)			Age group in female N (thousands), (%)			Sex N (thousands), (%)	
		18-24 years	25-60 years	> 60 years	18-24 years	25-60 years	> 60 years	Male	Female
Brazil	160 097	11 235 (7.0)	52 893 (33.0)	13 754 (8.6)	10 922 (6.8)	54 358 (34.0)	16 935 (10.6)	77 882 (48.6)	82 215 (51.4)
Ecuador	12 538	1 112 (8.9)	4 182 (33.4)	893 (7.1)	1 075 (8.6)	4 214 (33.6)	1 062 (8.5)	6 188 (49.3)	6 351 (50.7)
Georgia	2 869	153 (5.3)	851 (29.7)	287 (10.0)	144 (5.0)	944 (32.9)	490 (17.1)	1 291 (45.0)	1 578 (55.0)
Germany	70 490	3 034 (4.3)	20 483 (29.1)	11 007 (15.6)	2 807 (4.0)	19 858 (28.2)	13 301 (18.9)	34 524 (49.0)	35 966 (51.0)
India	1 001 432	95 215 (9.5)	351 295 (35.1)	67 524 (6.7)	86 362 (8.6)	328 546 (32.8)	72 490 (7.2)	514 034 (51.3)	487 398 (48.7)
Iran	66 100	4 181 (6.3)	24 409 (36.9)	4 876 (7.4)	3 990 (6.0)	23 552 (35.6)	5 092 (7.7)	33 467 (50.6)	32 634 (49.4)
Japan	106 514	4 296 (4.0)	28 050 (26.3)	19 181 (18.0)	4 078 (3.8)	27 320 (25.6)	23 589 (22.1)	51 527 (48.4)	54 986 (51.6)
Kuwait	3 767	174 (4.6)	2 103 (55.8)	133 (3.5)	160 (4.2)	1 094 (29.0)	103 (2.7)	2 410 (64.0)	1 356 (36.0)
North Macedonia	1 458	75 (5.1)	441 (30.3)	184 (12.6)	73 (5.0)	450 (30.9)	234 (16.1)	700 (48.0)	758 (52.0)
Oman	3 602	304 (8.4)	1 991 (55.3)	101 (2.8)	193 (5.4)	914 (25.4)	99 (2.7)	2 396 (66.5)	1 205 (33.5)
Peru	23 859	1 971 (8.3)	7 894 (33.1)	1 890 (7.9)	1 952 (8.2)	8 013 (33.6)	2 139 (9.0)	11 755 (49.3)	12 103 (50.7)
Poland	31 700	1 334 (4.2)	9 834 (31.0)	3 991 (12.6)	1 276 (4.0)	9 680 (30.5)	5 585 (17.6)	15 160 (47.8)	16 540 (52.2)
Portugal	8 777	396 (4.5)	2 383 (27.1)	1 344 (15.3)	378 (4.3)	2 528 (28.8)	1 748 (19.9)	4 123 (47.0)	4 654 (53.0)
Russia	115 259	5 156 (4.5)	35 312 (30.6)	11 579 (10.0)	4 958 (4.3)	37 539 (32.6)	20 715 (18.0)	52 048 (45.2)	63 211 (54.8)
Saudi Arabia	23 809	1 934 (8.1)	12 566 (52.8)	856 (3.6)	1 551 (6.5)	6 271 (26.3)	631 (2.6)	15 355 (64.5)	8 452 (35.5)
South Korea	44 704	2 042 (4.6)	14 369 (32.1)	5 801 (13.0)	1 876 (4.2)	13 540 (30.3)	7 076 (15.8)	22 212 (49.7)	22 493 (50.3)
Spain	40 030	1 767 (4.4)	12 148 (30.3)	5 553 (13.9)	1 664 (4.2)	11 997 (30.0)	6 901 (17.2)	19 468 (48.6)	20 562 (51.4)
Thailand	58 320	3 313 (5.7)	18 457 (31.6)	6 295 (10.8)	3 147 (5.4)	19 198 (32.9)	7 910 (13.6)	28 064 (48.1)	30 255 (51.9)
Türkiye	64 428	4 758 (7.4)	21 863 (33.9)	5 320 (8.3)	4 560 (7.1)	21 500 (33.4)	6 427 (10.0)	31 940 (49.6)	32 487 (50.4)

Supplementary Table S3.1. Prevalence of each type of dermographism of all participants in Ecuador (n=3582)

Age group	Ecuador population ≥ 18 years in 2023[‡] N (%)	Sample in this study n (%)	Point prevalence						Lifetime prevalence					
			Physiological red dermographism		Simple urticarial dermographism		Symptomatic dermographism		Physiological red dermographism		Simple urticarial dermographism		Symptomatic dermographism	
			n/N	% (95% CI)	n/N	% (95% CI)	n/N	% (95% CI)	n/N	% (95% CI)	n/N	% (95% CI)	n/N	% (95% CI)
**Male**														
18-24 years	1 112 000 (8.9)	454 (12.7)	67/454	14.76 (11.44-18.74)	28/454	6.17 (4.10-8.91)	8/454	1.76 (0.76-3.47)	270/454	59.47 (52.59-67.00)	130/454	28.63 (23.92-34.00)	45/454	9.91 (7.23-13.26)
25-60 years	4 182 000 (33.4)	497 (13.9)	55/497	11.07 (8.34-14.40)	12/497	2.41 (1.25-4.22)	12/497	2.41 (1.25-4.22)	253/497	50.91 (44.83-57.58)	107/497	21.53 (17.64-26.02)	46/497	9.26 (6.78-12.35)
> 60 years	893 000 (7.1)	484 (13.5)	78/484	16.12 (12.74-20.11)	15/484	3.10 (1.74-5.11)	18/484	3.72 (2.20-5.88)	250/484	51.65 (45.45-58.47)	80/484	16.53 (13.11-20.57)	59/484	12.19 (9.28-15.72)
% Crude prevalence (95% CI)			200/1435	13.94 (12.07-16.01)	55/1435	3.83 (2.89-4.99)	38/1435	2.65 (1.87-3.64)	773/1435	53.87 (50.14-57.80)	317/1435	22.09 (19.73-24.66)	150/1435	10.45 (8.85-12.27)
% Age-adjusted prevalence (95% CI)				12.46 (10.45-14.47)		3.19 (2.17-4.20)		2.48 (1.51-3.45)		52.56 (49.41-55.70)		22.08 (19.49-24.68)		9.80 (7.96-11.64)
**Female**														
18-24 years	1 075 000 (8.6)	746 (20.8)	172/746	23.06 (19.74-26.77)	54/746	7.24 (5.44-9.45)	22/746	2.95 (1.85-4.47)	541/746	72.52 (66.54-78.90)	266/746	35.66 (31.50-40.21)	82/746	10.99 (8.74-13.64)
25-60 years	4 214 000 (33.6)	767 (21.4)	102/767	13.30 (10.84-16.14)	23/767	3.00 (1.90-4.50)	33/767	4.30 (2.96-6.04)	476/767	62.06 (56.61-67.89)	206/767	26.86 (23.32-30.79)	93/767	12.13 (9.79-14.85)
> 60 years	1 062 000 (8.5)	634 (17.7)	91/634	14.35 (11.56-17.62)	13/634	2.05 (1.09-3.51)	19/634	3.00 (1.80-4.68)	324/634	51.10 (45.69-56.98)	91/634	14.35 (11.56-17.62)	68/634	10.73 (8.33-13.60)
% Crude prevalence (95% CI)			365/2147	17.00 (15.30-18.84)	90/2147	4.19 (3.37-5.15)	74/2147	3.45 (2.71-4.33)	1341/2147	62.46 (59.16-65.89)	563/2147	26.22 (24.10-28.48)	243/2147	11.32 (9.94-12.83)
% Age-adjusted prevalence (95% CI)				15.13 (13.39-16.86)		3.56 (2.68-4.44)		3.85 (2.86-4.85)		62.00 (59.57-64.43)		26.26 (24.05-28.47)		11.70 (10.07-13.33)
**Total**	12 538 000 (100.0)	3582 (100.0)	565/3582	15.77 (14.50-17.13)	145/3582	4.05 (3.42-4.76)	112/3582	3.13 (2.58-3.76)	2114/3582	59.02 (56.53-61.59)	880/3582	24.57 (22.97-26.25)	393/3582	10.97 (9.91-12.11)
**% Crude prevalence (95% CI)**				**15.77 (14.50-17.13)**		**4.05 (3.42-4.76)**		**3.13 (2.58-3.76)**		**59.02 (56.53-61.59)**		**24.57 (22.97-26.25)**		**10.97 (9.91-12.11)**
**% Sex- and age-adjusted prevalence[†] (95% CI)**				**13.81 (12.49-15.14)**		**3.38 (2.70-4.05)**		**3.18 (2.48-3.87)**		**57.34 (55.36-59.32)**		**24.20 (22.50-25.90)**		**10.76 (9.54-11.99)**

Supplementary Table S3.2. Prevalence of each type of dermographism of all participants in Germany (n=3541)

Age group	Germany population ≥ 18 years in 2023‡ N (%)	Sample in this study n (%)	Point prevalence						Lifetime prevalence					
			Physiological red dermographism		Simple urticarial dermographism		Symptomatic dermographism		Physiological red dermographism		Simple urticarial dermographism		Symptomatic dermographism	
			n/N	% (95% CI)	n/N	% (95% CI)	n/N	% (95% CI)	n/N	% (95% CI)	n/N	% (95% CI)	n/N	% (95% CI)
**Male**														
18-24 years	3 034 000 (4.3)	270 (7.6)	36/270	13.33 (9.34-18.46)	5/270	1.85 (0.60-4.32)	13/270	4.81 (2.56-8.23)	162/270	60.00 (51.12-69.98)	63/270	23.33 (17.93-29.85)	30/270	11.11 (7.50-15.86)
25-60 years	20 483 000 (29.1)	269 (7.6)	71/269	26.39 (20.61-33.29)	4/269	1.49 (0.41-3.81)	25/269	9.29 (6.01-13.72)	160/269	59.48 (50.62-69.44)	37/269	13.75 (9.68-18.96)	34/269	12.64 (8.75-17.66)
> 60 years	11 007 000 (15.6)	645 (18.2)	30/645	4.65 (3.14-6.64)	3/645	0.47 (0.10-1.36)	9/645	1.40 (0.64-2.65)	213/645	33.02 (28.74-37.77)	73/645	11.32 (8.87-14.23)	30/645	4.65 (3.14-6.64)
% Crude prevalence (95% CI)			137/1184	11.57 (9.71-13.68)	12/1184	1.01 (0.53-1.77)	47/1184	3.97 (2.92-5.28)	535/1184	45.19 (41.44-49.18)	173/1184	14.61 (12.52-16.96)	94/1184	7.94 (6.42-9.72)
% Age-adjusted prevalence (95% CI)				18.31 (15.12-21.50)		1.20 (0.31-2.08)		6.38 (4.29-8.47)		51.09 (47.39-54.79)		13.82 (11.22-16.42)		9.96 (7.52-12.39)
**Female**														
18-24 years	2 807 000 (4.0)	972 (27.4)	193/972	19.86 (17.15-22.86)	28/972	2.88 (1.91-4.16)	38/972	3.91 (2.77-5.37)	712/972	73.25 (67.97-78.83)	331/972	34.05 (30.48-37.93)	103/972	10.60 (8.65-12.85)
25-60 years	19 858 000 (28.2)	835 (23.6)	240/835	28.74 (25.22-32.62)	32/835	3.83 (2.62-5.41)	101/835	12.10 (9.85-14.70)	600/835	71.86 (66.22-77.84)	168/835	20.12 (17.19-23.40)	133/835	15.93 (13.34-18.88)
> 60 years	13 301 000 (18.9)	550 (15.5)	43/550	7.82 (5.66-10.53)	5/550	0.91 (0.30-2.12)	14/550	2.55 (1.39-4.27)	237/550	43.09 (37.78-48.94)	80/550	14.55 (11.53-18.10)	37/550	6.73 (4.74-9.27)
% Crude prevalence (95% CI)			476/2357	20.20 (18.42-22.09)	65/2357	2.76 (2.13-3.51)	153/2357	6.49 (5.50-7.61)	1549/2357	65.72 (62.49-69.08)	579/2357	24.57 (22.60-26.65)	273/2357	11.58 (10.25-13.04)
% Age-adjusted prevalence (95% CI)				20.31 (18.21-22.21)		2.68 (1.90-3.46)		7.93 (6.61-9.25)		61.33 (59.04-63.62)		19.15 (17.28-21.02)		12.11 (10.53-13.69)
**Total**	70 490 000 (100.0)	3541 (100.0)	613/3541	17.31 (15.97-18.74)	77/3541	2.17 (1.72-2.72)	200/3541	5.65 (4.89-6.49)	2084/3541	58.85 (56.35-61.44)	752/3541	21.24 (19.75-22.81)	367/3541	10.36 (9.33-11.48)
**% Crude prevalence (95% CI)**				17.31 (15.97-18.74)		2.17 (1.72-2.72)		5.65 (4.89-6.49)		58.85 (56.35-61.44)		21.24 (19.75-22.81)		10.36 (9.33-11.48)
**% Sex- and age-adjusted prevalence† (95% CI)**				**19.33 (17.49-21.17)**		**1.95 (1.36-2.54)**		**7.17 (5.95-8.40)**		**56.31 (54.16-58.47)**		**16.54 (14.95-18.13)**		**11.06 (9.62-12.50)**

Supplementary Table S3.3. Prevalence of each type of dermographism of all participants in India (n=3092)

Age group	India population ≥ 18 years in 2023[‡] N (%)	Sample in this study n (%)	Point prevalence						Lifetime prevalence					
			Physiological red dermographism		Simple urticarial dermographism		Symptomatic dermographism		Physiological red dermographism		Simple urticarial dermographism		Symptomatic dermographism	
			n/N	% (95% CI)	n/N	% (95% CI)	n/N	% (95% CI)	n/N	% (95% CI)	n/N	% (95% CI)	n/N	% (95% CI)
**Male**														
18-24 years	95 215 000 (9.5)	384 (14.4)	24/384	6.25 (4.00-9.30)	5/384	1.30 (0.42-3.04)	7/384	1.82 (0.73-3.76)	42/384	10.94 (7.88-14.78)	17/384	4.43 (2.58-7.09)	11/384	2.86 (1.43-5.13)
25-60 years	351 295 000 (35.1)	377 (19.5)	8/377	2.12 (0.92-4.18)	0/377	0.00 N/A	11/377	2.92 (1.46-5.22)	141/377	37.40 (31.48-44.11)	4/377	1.06 (0.29-2.72)	15/377	3.98 (2.23-6.56)
> 60 years	67 524 000 (6.7)	447 (17.4)	35/447	7.83 (5.45-10.89)	0/447	0.00 N/A	2/447	0.45 (0.05-1.62)	63/447	14.09 (10.83-18.03)	0/447	0.00 N/A	3/447	0.67 (0.14-1.96)
% Crude prevalence (95% CI)			67/1208	5.55 (4.30-7.04)	5/1208	0.41 (0.13-0.97)	20/1208	1.66 (1.01-2.56)	246/1208	20.36 (17.90-23.07)	21/1208	1.74 (1.08-2.66)	29/1208	2.40 (1.61-3.45)
% Age-adjusted prevalence (95% CI)				3.64 (2.50-4.77)		1.30 (0.17-2.43)		2.39 (1.20-3.58)		29.44 (26.03-32.85)		1.78 (0.85-2.70)		3.34 (1.95-4.73)
**Female**														
18-24 years	86 362 000 (8.6)	620 (17.3)	33/620	5.32 (3.66-7.48)	5/620	0.81 (0.26-1.88)	8/620	1.29 (0.56-2.54)	86/620	13.87 (11.09-17.13)	40/620	6.45 (4.61-8.79)	16/620	2.58 (1.48-4.19)
25-60 years	328 546 000 (32.8)	709 (17.7)	12/709	1.69 (0.88-2.96)	1/709	0.14 (0.00-0.79)	19/709	2.68 (1.61-4.19)	161/709	22.71 (19.34-26.50)	9/709	1.27 (0.58-2.41)	25/709	3.53 (2.28-5.21)
> 60 years	72 490 000 (7.2)	555 (13.7)	80/555	14.41 (11.43-17.94)	0/555	0.00 N/A	3/555	0.54 (0.11-1.58)	194/555	34.95 (30.21-40.23)	3/555	0.54 (0.11-1.58)	3/555	0.54 (0.11-1.58)
% Crude prevalence (95% CI)			125/1884	6.63 (5.52-7.91)	6/1884	0.32 (0.12-0.69)	30/1884	1.59 (1.07-2.27)	441/1884	23.41 (21.27-25.70)	52/1884	2.76 (2.06-3.62)	44/1884	2.34 (1.70-3.14)
% Age-adjusted prevalence (95% CI)				4.23 (3.39-5.06)		0.28 (0.02-0.54)		2.12 (1.29-2.94)		22.96 (20.75-25.18)		2.08 (1.42-2.74)		2.92 (1.97-3.86)
**Total**	1 001 432 000 (100.0)	3092 (100.0)	192/3092	6.21 (5.36-7.15)	11/3092	0.36 (0.18-0.64)	50/3092	1.62 (1.20-2.13)	687/3092	22.22 (20.59-23.94)	73/3092	2.36 (1.85-2.97)	73/3092	2.36 (1.85-2.97)
**% Crude prevalence (95% CI)**				6.21 (5.36-7.15)		0.36 (0.18-0.64)		1.62 (1.20-2.13)		22.22 (20.59-23.94)		2.36 (1.85-2.97)		2.36 (1.85-2.97)
**% Sex- and age-adjusted prevalence[†] (95% CI)**				**3.92 (3.21-4.63)**		**0.47 (0.17-0.77)**		**2.26 (1.53-2.99)**		**26.87 (24.23-28.34)**		**1.94 (1.38-2.50)**		**3.13 (2.29 -3.98)**

Supplementary Table S3.4. Prevalence of each type of dermographism of all participants in Iran (n=3253)

Age group	Iran population ≥ 18 years in 2023[‡] N (%)	Sample in this study n (%)	Point prevalence						Lifetime prevalence					
			Physiological red dermographism		Simple urticarial dermographism		Symptomatic dermographism		Physiological red dermographism		Simple urticarial dermographism		Symptomatic dermographism	
			n/N	% (95% CI)	n/N	% (95% CI)	n/N	% (95% CI)	n/N	% (95% CI)	n/N	% (95% CI)	n/N	% (95% CI)
**Male**														
18-24 years	4 181 000 (6.3)	516 (15.9)	19/516	3.68 (2.22-5.75)	14/516	2.71 (1.48-4.55)	3/516	0.58 (0.12-1.70)	81/516	15.70 (12.47-19.51)	38/516	7.36 (5.21-10.11)	5/516	0.97 (0.31-2.26)
25-60 years	24 409 000 (36.9)	521 (16.0)	25/521	4.80 (3.11-7.08)	12/521	2.30 (1.19-4.02)	3/521	0.58 (0.12-1.68)	136/521	26.10 (21.9-30.88)	33/521	6.33 (4.36-8.90)	10/521	1.92 (0.92-3.53)
> 60 years	4 876 000 (7.4)	452 (13.9)	10/452	2.21 (1.06-4.07)	10/452	2.21 (1.06-4.07)	1/452	0.22 (0.01-1.23)	67/452	14.82 (11.49-18.82)	37/452	8.19 (5.76-11.28)	6/452	1.33 (0.49-2.89)
% Crude prevalence (95% CI)			54/1489	3.63 (2.72-4.73)	36/1489	2.42 (1.69-3.35)	7/1489	0.47 (0.19-0.97)	284/1489	19.07 (16.92-21.43)	108/1489	7.25 (5.95-8.76)	21/1489	1.41 (0.87-2.16)
% Age-adjusted prevalence (95% CI)				4.28 (2.91-5.65)		2.34 (1.36-3.31)		0.53 (0.04-1.01)		23.16 (20.34-25.98)		6.73 (5.14-8.32)		1.72 (0.84-2.60)
**Female**														
18-24 years	3 990 000 (6.0)	515 (15.8)	29/515	5.63 (3.77-8.09)	9/515	1.75 (0.80-3.32)	3/515	0.58 (0.12-1.70)	76/515	14.76 (11.63-18.47)	37/515	7.18 (5.06-9.90)	9/515	1.75 (0.80-3.32)
25-60 years	23 552 000 (35.6)	701 (21.5)	36/701	5.14 (3.60-7.11)	10/701	1.43 (0.68-2.62)	8/701	1.14 (0.49-2.25)	145/701	20.68 (17.46-24.34)	34/701	4.85 (3.36-6.78)	22/701	3.14 (1.97-4.75)
> 60 years	5 092 000 (7.7)	548 (16.8)	22/548	4.01 (2.52-6.08)	11/548	2.01 (1.00-3.59)	1/548	0.18 (0.00-1.02)	106/548	19.34 (15.84-23.39)	29/548	5.29 (3.54-7.60)	4/548	0.73 (0.20-1.87)
% Crude prevalence (95% CI)			87/1764	4.93 (3.95-6.08)	30/1764	1.70 (1.15-2.43)	12/1764	0.68 (0.35-1.19)	327/1764	18.54 (16.58-20.66)	100/1764	5.67 (4.61-6.90)	35/1764	1.98 (1.38-2.76)
% Age-adjusted prevalence (95% CI)				5.02 (3.79-6.26)		1.56 (0.89-2.23)		0.92 (0.35-1.50)		19.75 (17.49-22.00)		5.20 (3.99-6.42)		2.59 (1.65-3.54)
**Total**	66 100 000 (100.0)	3253 (100.0)	141/3253	4.33 (3.65-5.11)	66/3253	2.03 (1.57-2.58)	19/3253	0.58 (0.35-0.91)	611/3253	18.78 (17.32-20.33)	208/3253	6.39 (5.56-7.33)	56/3253	1.72 (1.30-2.24)
**% Crude prevalence (95% CI)**				**4.33 (3.65-5.11)**		**2.03 (1.57-2.58)**		**0.58 (0.35-0.91)**		**18.78 (17.32-20.33)**		**6.39 (5.56-7.33)**		**1.72 (1.30-2.24)**
**% Sex- and age-adjusted prevalence[†] (95% CI)**				**4.65 (3.73-5.57)**		**1.95 (1.36-2.55)**		**0.72 (0.35-1.10)**		**21.47 (19.66-23.28)**		**5.98 (4.97-6.98)**		**2.15 (1.50-2.80)**

Supplementary Table S3.5. Prevalence of each type of dermographism of all participants in Kuwait (n=3556)

Age group	Kuwait population ≥ 18 years in 2023[‡] N (%)	Sample in this study n (%)	Point prevalence						Lifetime prevalence					
			Physiological red dermographism		Simple urticarial dermographism		Symptomatic dermographism		Physiological red dermographism		Simple urticarial dermographism		Symptomatic dermographism	
			n/N	% (95% CI)	n/N	% (95% CI)	n/N	% (95% CI)	n/N	% (95% CI)	n/N	% (95% CI)	n/N	% (95% CI)
**Male**														
18-24 years	174 000 (4.6)	427 (12.0)	20/427	4.68 (2.86-7.23)	23/427	5.39 (3.42-8.08)	7/427	1.64 (0.66-3.38)	80/427	18.74 (14.86-23.32)	98/427	22.95 (18.63-27.97)	22/427	5.15 (3.23-7.80)
25-60 years	2 103 000 (55.8)	630 (17.7)	35/630	5.56 (3.87-7.73)	8/630	1.27 (0.55-2.50)	12/630	1.91 (0.98-3.33)	114/630	18.10 (14.93-21.74)	43/630	6.83 (4.94-9.19)	28/630	4.44 (2.95-6.42)
> 60 years	133 000 (3.5)	567 (15.9)	17/567	2.99 (1.75-4.80)	2/567	0.35 (0.04-1.27)	13/567	2.29 (1.22-3.92)	77/567	13.58 (10.72-16.97)	32/567	5.64 (3.86-7.97)	21/567	3.70 (2.29-5.66)
% Crude prevalence (95% CI)			72/1624	4.43 (3.47-5.58)	33/1624	2.03 (1.39-2.85)	32/1624	1.97 (1.35-2.78)	271/1624	16.69 (14.76-18.80)	173/1624	10.65 (9.12-12.36)	71/1624	4.37 (3.42-5.52)
% Age-adjusted prevalence (95% CI)				5.35 (3.79-6.92)		1.52 (0.74-2.30)		1.91 (0.97-2.85)		17.90 15.26-20.54)		7.93 (6.18-9.67)		4.45 (3.04-5.86)
**Female**														
18-24 years	160 000 (4.2)	618 (17.4)	46/618	7.44 (5.45-9.93)	41/618	6.63 (4.76-9.00)	11/618	1.78 (0.89-3.18)	128/618	20.71 (17.28-24.63)	130/618	21.04 (17.58-24.98)	27/618	4.37 (2.88-6.36)
25-60 years	1 094 000 (29.0)	836 (23.5)	83/836	9.93 (7.91-12.31)	20/836	2.39 (1.46-3.69)	44/836	5.26 (3.82-7.07)	245/836	29.31 (25.75-33.21)	90/836	10.77 (8.66-13.23)	82/836	9.81 (7.80-12.18)
> 60 years	103 000 (2.7)	478 (13.5)	32/478	6.69 (4.58-9.45)	9/478	1.88 (0.86-3.57)	13/478	2.72 (1.45-4.65)	69/478	14.44 (11.23-18.27)	30/478	6.28 (4.24-8.96)	29/478	6.07 (4.06-8.71)
% Crude prevalence (95% CI)			161/1932	8.33 (7.10-9.73)	70/1932	3.62 (2.82-4.58)	68/1932	3.52 (2.73-4.46)	442/1932	22.88 (20.79-25.11)	250/1932	12.94 (11.39-14.65)	138/1932	7.14 (6.00-8.44)
% Age-adjusted prevalence (95% CI)				9.39 (7.73-11.05)		2.85 (1.98-3.72)		4.66 (3.42-5.89)		27.17 (24.64-29.69)		11.64 (9.90-13.38)		8.86 (7.24-10.53)
**Total**	3 767 000 (100.0)	3556 (100.0)	233/3556	6.55 (5.74-7.45)	103/3556	2.89 (2.36-3.51)	100/3556	2.81 (2.29-3.42)	713/3556	20.05 (18.61-21.58)	423/3556	11.90 (10.79-13.08)	209/3556	5.88 (5.11-6.73)
**% Crude prevalence (95% CI)**			**233/3556**	**6.55 (5.74-7.45)**	**103/3556**	**2.89 (2.36-3.51)**	**100/3556**	**2.81 (2.29-3.42)**	**713/3556**	**20.05 (18.61-21.58)**	**423/3556**	**11.90 (10.79-13.08)**	**209/3556**	**5.88 (5.11-6.73)**
**% Sex- and age-adjusted prevalence[†] (95% CI)**				**6.81 (5.64-7.98)**		**2.00 (1.41-2.59)**		**2.90 (2.15-3.65)**		**21.23 (19.32-23.10)**		**9.27 (7.98-10.55)**		**6.05 (4.97-7.13)**

Supplementary Table S3.6. Prevalence of each type of dermographism of all participants in Oman (n=7106)

Age group	Oman population ≥ 18 years in 2023[‡] N (%)	Sample in this study n (%)	Point prevalence						Lifetime prevalence					
			Physiological red dermographism		Simple urticarial dermographism		Symptomatic dermographism		Physiological red dermographism		Simple urticarial dermographism		Symptomatic dermographism	
			n/N	% (95% CI)	n/N	% (95% CI)	n/N	% (95% CI)	n/N	% (95% CI)	n/N	% (95% CI)	n/N	% (95% CI)
**Male**														
18-24 years	304 000 (8.4)	949 (13.4)	636/949	67.02 (61.91-72.43)	5/949	0.53 (0.17-1.23)	5/949	0.53 (0.02-1.23)	653/949	68.81 (63.63-74.30)	14/949	1.48 (0.81-2.48)	13/949	1.37 (0.73-2.34)
25-60 years	1 991 000 (55.3)	1280 (18.0)	753/1280	58.83 (54.70-63.18)	8/1280	0.63 (0.27-1.23)	8/1280	0.63 (0.27-1.23)	777/1280	60.70 (56.51-65.13)	41/1280	3.20 (2.30-4.35)	31/1280	2.42 (1.65-3.44)
> 60 years	101 000 (2.8)	1160 (16.3)	192/1160	16.55 (14.29-19.07)	2/1160	0.17 (0.02-0.62)	1/1160	0.09 (0.00-0.48)	195/1160	16.81 (14.53-19.34)	10/1160	0.86 (0.41-1.59)	1/1160	0.09 (0.00-0.48)
% Crude prevalence (95% CI)			1581/3389	46.65 (44.38-49.01)	15/3389	0.44 (0.25-0.73)	14/3389	0.41 (0.23-0.69)	1625/3389	47.95 (45.65-50.34)	65/3389	1.92 (1.48-2.45)	45/3389	1.33 (0.97-1.78)
% Age-adjusted prevalence (95% CI)				58.09 (55.81-60.36)		0.60 (0.23-0.96)		0.60 (0.23-0.96)		59.89 (57.62-61.13)		2.88 (2.08-3.69)		2.19 (1.48-2.89)
**Female**														
18-24 years	193 000 (5.4)	1092 (15.4)	737/1092	67.49 (62.71-72.54)	25/1092	2.29 (1.48-3.38)	15/1092	1.37 (0.77-2.27)	791/1092	72.44 (67.48-77.66)	41/1092	3.75 (2.69-5.09)	28/1092	2.56 (1.70-3.71)
25-60 years	914 000 (25.4)	1721 (24.2)	1019/1721	59.21 (55.63-62.96)	35/1721	2.03 (1.42-2.83)	72/1721	4.18 (3.27-5.27)	1168/1721	67.87 (64.03-71.87)	136/1721	7.90 (6.63-9.35)	116/1721	6.74 (5.57-8.08)
> 60 years	99 000 (2.7)	904 (12.7)	203/904	22.46 (19.47-25.77)	3/904	0.33 (0.07-0.97)	0/904	0.00 (N/A)	210/904	23.23 (20.19-26.59)	5/904	0.55 (0.18-1.29)	2/904	0.22 (0.03-0.80)
% Crude prevalence (95% CI)			1959/3717	52.7 (50.40-55.09)	63/3717	1.69 (1.30-2.17)	87/3717	2.34 (1.88-2.89)	2169/3717	58.35 (55.92-60.86)	182/3717	4.90 (4.21-5.66)	146/3717	3.93 (3.32-4.62)
% Age-adjusted prevalence (95% CI)				57.52 (55.69-59.35)		1.93 (1.41-2.46)		3.69 (2.90-4.48)		64.94 (63.20-66.68)		6.63 (5.65-7.62)		5.54 (4.63-6.45)
**Total**	3 602 000 (100.0)	7106 (100.0)	3540/7106	49.82 (48.19-51.49)	78/7106	1.10 (0.87-1.37)	101/7106	1.42 (1.16-1.73)	3794/7106	53.39 (51.71-55.12)	247/7106	3.48 (3.06-3.94)	191/7106	2.69 (2.32-3.10)
**% Crude prevalence (95% CI)**				**49.82 (48.19-51.49)**		**1.10 (0.87-1.37)**		**1.42 (1.16-1.73)**		**53.39 (51.71-55.12)**		**3.48 (3.06-3.94)**		**2.69 (2.32-3.10)**
**% Sex- and age-adjusted prevalence[†] (95% CI)**				**57.90 (56.27-59.53)**		**1.06 (0.75-1.34)**		**1.57 (1.22-1.93)**		**61.57 (59.96-63.18)**		**4.14 (3.51-4.77)**		**3.31 (2.75-3.87)**

Supplementary Table S3.7. Prevalence of each type of dermographism of all participants in Peru (n=6447)

Age group	Peru population ≥ 18 years in 2023[‡] N (%)	Sample in this study n (%)	Point prevalence						Lifetime prevalence					
			Physiological red dermographism		Simple urticarial dermographism		Symptomatic dermographism		Physiological red dermographism		Simple urticarial dermographism		Symptomatic dermographism	
			n/N	% (95% CI)	n/N	% (95% CI)	n/N	% (95% CI)	n/N	% (95% CI)	n/N	% (95% CI)	n/N	% (95% CI)
**Male**														
18-24 years	1 971 000 (8.3)	927 (14.4)	52/927	5.61 (4.19-7.36)	7/927	0.76 (0.30-1.56)	39/927	4.21 (2.99-5.75)	374/927	40.35 (36.36-44.65)	98/927	10.57 (8.58-12.88)	130/927	14.02 (11.72-16.65)
25-60 years	7 894 000 (33.1)	1259 (19.5)	91/1259	7.23 (5.82-8.87)	21/1259	1.67 (1.03-2.55)	14/1259	1.11 (0.61-1.87)	561/1259	44.56 (40.95-48.40)	147/1259	11.68 (9.86-13.72)	99/1259	7.86 (6.39-9.57)
> 60 years	1 890 000 (7.9)	1119 (17.4)	51/1119	4.56 (3.39-5.99)	8/1119	0.71 (0.31-1.41)	22/1119	1.97 (1.23-2.98)	455/1119	40.66 (37.01-44.57)	128/1119	11.44 (9.54-13.60)	167/1119	14.92 (12.75-17.37)
% Crude prevalence (95% CI)			194/3305	5.87 (5.07-6.76)	36/3305	1.09 (0.76-1.51)	75/3305	2.27 (1.79-2.85)	1390/3305	42.06 (39.88-44.33)	373/3305	11.29 (10.17-12.49)	396/3305	11.98 (10.83-13.22)
% Age-adjusted prevalence (95% CI)				6.53 (5.52-7.54)		1.36 (0.87-1.85)		1.77 (1.30-2.23)		43.23 (41.25-45.20)		11.46 (10.18-12.73)		10.03 (8.91-11.15)
**Female**														
18-24 years	1 952 000 (8.2)	1118 (17.3)	90/1118	8.05 (6.47-9.90)	33/1118	2.95 (2.03-4.15)	27/1118	2.42 (1.59-3.51)	399/1118	35.69 (32.27-39.37)	248/1118	22.18 (19.51-25.12)	57/1118	5.10 (3.86-6.61)
25-60 years	8 013 000 (33.6)	1139 (17.7)	137/1139	12.03 (10.10-14.22)	48/1139	4.21 (3.11-5.59)	22/1139	1.93 (1.21-2.92)	474/1139	41.62 (37.95-45.54)	205/1139	18.00 (15.62-20.64)	83/1139	7.29 (5.80-9.03)
> 60 years	2 139 000 (9.0)	885 (13.7)	72/885	8.14 (6.37-10.25)	32/885	3.62 (2.47-5.10)	19/885	2.15 (1.29-3.35)	255/885	28.81 (25.39-32.58)	169/885	19.10 (16.33-22.20)	27/885	3.05 (2.01-4.44)
% Crude prevalence (95% CI)			299/3142	9.52 (8.47-10.66)	113/3142	3.60 (2.96-4.32)	68/3142	2.16 (1.68-2.74)	1128/3142	35.90 (33.84-38.06)	622/3142	19.80 (18.27-21.42)	167/3142	5.32 (4.54-6.19)
% Age-adjusted prevalence (95% CI)				10.70 (9.39-12.02)		3.90 (3.09-4.72)		2.05 (1.47-2.62)		38.40 (36.38-40.42)		18.87 (17.27-20.46)		6.19 (5.15-7.23)
**Total**	23 859 000 (100.0)	6447 (100.0)	493/6447	7.65 (6.99-8.35)	149/6447	2.31 (1.96-2.71)	143/6447	2.22 (1.87-2.61)	2518/6447	39.06 (37.55-40.61)	995/6447	15.43 (14.49-16.42)	563/6447	8.73 (8.03-9.49)
**% Crude prevalence (95% CI)**				**7.65 (6.99-8.35)**		**2.31 (1.96-2.71)**		**2.22 (1.87-2.61)**		**39.06 (37.55-40.61)**		**15.43 (14.49-16.42)**		**8.73 (8.03-9.49)**
**% Sex- and age-adjusted prevalence[†] (95% CI)**				**8.65 (7.81-9.48)**		**2.65 (2.17-3.13)**		**1.91 (1.54-2.28)**		**40.78 (39.37-42.19)**		**15.22 (14.19-16.24)**		**8.08 (7.32-8.84)**

Supplementary Table S3.8. Prevalence of each type of dermographism of all participants in Poland (n=12 084)

Age group	Poland population ≥ 18 years in 2023[‡] N (%)	Sample in this study n (%)	Point prevalence						Lifetime prevalence					
			Physiological red dermographism		Simple urticarial dermographism		Symptomatic dermographism		Physiological red dermographism		Simple urticarial dermographism		Symptomatic dermographism	
			n/N	% (95% CI)	n/N	% (95% CI)	n/N	% (95% CI)	n/N	% (95% CI)	n/N	% (95% CI)	n/N	% (95% CI)
**Male**														
18-24 years	1 334 000 (4.2)	1040 (8.6)	374/1040	35.96 (32.41-39.80)	28/1040	2.69 (1.79-3.89)	23/1040	2.21 (1.40-3.32)	469/1040	45.10 (41.11-49.37)	74/1040	7.12 (5.59-8.93)	37/1040	3.56 (2.51-4.90)
25-60 years	9 834 000 (31.0)	2062 (17.0)	523/2062	25.36 (23.24-27.63)	48/2062	2.33 (1.72-3.09)	69/2062	3.35 (2.60-4.24)	692/2062	33.56 (31.11-36.16)	104/2062	5.04 (4.12-6.11)	122/2062	5.92 (4.91-7.06)
> 60 years	3 991 000 (12.6)	1059 (8.8)	155/1059	14.64 (12.42-17.13)	10/1059	0.94 (0.45-1.74)	8/1059	0.76 (0.33-1.49)	229/1059	21.62 (18.91-24.61)	20/1059	1.89 (1.15-2.92)	20/1059	1.89 (1.15-2.92)
% Crude prevalence (95% CI)			1052/4161	25.28 (23.78-26.86)	86/4161	2.07 (1.65-2.55)	100/4161	2.40 (1.96-2.92)	1390/4161	33.41 (31.67-35.21)	198/4161	4.76 (4.12-5.47)	179/4161	4.30 (4.00-4.98)
% Age-adjusted prevalence (95% CI)				23.47 (22.11-24.84)		2.00 (1.54-2.45)		2.57 (2.04-3.10)		31.43 (29.93-32.93)		4.39 (3.73-5.06)		4.65 (3.95-5.35)
**Female**														
18-24 years	1 276 000 (4.0)	2401 (19.9)	1147/2401	47.77 (45.05-50.62)	126/2401	5.25 (4.37-6.25)	97/2401	4.04 (3.28-4.93)	1417/2401	59.02 (55.98-62.17)	322/2401	13.41 (11.99-14.96)	166/2401	6.91 (5.90-8.05)
25-60 years	9 680 000 (30.5)	4134 (34.2)	1585/4134	38.34 (36.48-40.28)	209/4134	5.06 (4.39-5.79)	235/4134	5.69 (4.98-6.46)	2075/4134	50.19 (48.06-52.40)	442/4134	10.69 (9.72-11.74)	398/4134	9.63 (8.71-10.62)
> 60 years	5 585 000 (17.6)	1388 (11.5)	357/1388	25.72 (23.12-28.53)	14/1388	1.01 (0.55-1.69)	22/1388	1.59 (0.99-2.40)	488/1388	35.16 (32.11-38.42)	41/1388	2.95 (2.12-4.01)	49/1388	3.53 (2.61-4.67)
% Crude prevalence (95% CI)			3089/7923	38.99 (37.62-40.39)	349/7923	4.41 (3.96-4.89)	354/7923	4.47 (4.02-4.96)	3980/7923	50.23 (48.68-51.82)	805/7923	10.16 (9.47-10.89)	613/7923	7.74 (7.14-8.34)
% Age-adjusted prevalence (95% CI)				34.81 (33.63-35.98)		3.71 (3.27-4.14)		4.18 (3.71-4.65)		45.78 (44.56-47.04)		8.29 (7.65-8.92)		7.36 (6.74-7.99)
**Total**	31 700 000 (100.0)	12 084 (100.0)	4141/12084	34.27 (33.23-35.33)	435/12084	3.60 (3.27-3.96)	454/12084	3.76 (3.42-4.12)	5370/12084	44.44 (43.26-45.64)	1003/12084	8.30 (7.78-8.83)	792/12084	6.55 (6.11-7.03)
**% Crude prevalence (95% CI)**				**34.27 (33.23-35.33)**		**3.60 (3.27-3.96)**		**3.76 (3.42-4.12)**		**44.44 (43.26-45.64)**		**8.30 (7.78-8.83)**		**6.55 (6.11-7.03)**
**% Sex- and age-adjusted prevalence[†] (95% CI)**				**29.39 (28.49-30.28)**		**2.89 (2.57-3.20)**		**3.41 (3.06-3.76)**		**38.93 (37.96-39.89)**		**6.43 (5.97-6.88)**		**6.07 (5.60-6.53)**

Supplementary Table S3.9. Prevalence of each type of dermographism of all participants in Portugal (n=4254)

Age group	Portugal population ≥ 18 years in 2023[‡] N (%)	Sample in this study n (%)	Point prevalence						Lifetime prevalence					
			Physiological red dermographism		Simple urticarial dermographism		Symptomatic dermographism		Physiological red dermographism		Simple urticarial dermographism		Symptomatic dermographism	
			n/N	% (95% CI)	n/N	% (95% CI)	n/N	% (95% CI)	n/N	% (95% CI)	n/N	% (95% CI)	n/N	% (95% CI)
**Male**														
18-24 years	396 000 (4.5)	301 (7.1)	49/301	16.28 (12.04-21.52)	10/301	3.32 (1.59-6.11)	11/301	3.65 (1.82-6.54)	150/301	49.83 (42.18-58.48)	48/301	15.95 (11.76-21.14)	28/301	9.30 (6.18-13.44)
25-60 years	2 383 000 (27.1)	614 (14.4)	69/614	11.24 (8.74-14.22)	9/614	1.47 (0.67-2.78)	24/614	3.91 (2.50-5.82)	290/614	47.23 (41.95-52.99)	114/614	18.57 (15.32-22.30)	54/614	8.79 (6.61-11.48)
> 60 years	1 344 000 (15.3)	367 (8.6)	27/367	7.36 (4.85-10.70)	5/367	1.36 (0.44-3.18)	8/367	2.18 (0.74-4.30)	125/367	34.06 (28.35-40.58)	30/367	8.17 (5.52-11.67)	26/367	7.08 (4.63-10.38)
% Crude prevalence (95% CI)			145/1282	11.31 (9.54-13.31)	24/1282	1.87 (1.20-2.79)	43/1282	3.35 (2.43-4.52)	565/1282	44.07 (40.51-47.86)	192/1282	14.98 (12.93-17.25)	108/1282	8.42 (6.91-10.17)
% Age-adjusted prevalence (95% CI)				10.46 (8.73-12.19)		1.61 (0.91-2.31)		3.32 (2.29-4.35)		43.19 (40.36-46.02)		14.93 (12.89-16.97)		8.28 (6.70-9.87)
**Female**														
18-24 years	378 000 (4.3)	700 (16.5)	171/700	24.43 (20.90-28.38)	41/700	5.86 (4.20-7.95)	42/700	6.00 (4.32-8.11)	444/700	63.43 (57.67-69.61)	205/700	29.29 (25.41-33.58)	89/700	12.71 (10.21-15.65)
25-60 years	2 528 000 (28.8)	1639 (38.5)	285/1639	17.39 (15.43-19.53)	56/1639	3.42 (2.58-4.44)	114/1639	6.96 (5.74-8.36)	1035/1639	63.15 (59.36-67.12)	379/1639	23.12 (20.85-25.57)	250/1639	15.25 (13.42-17.27)
> 60 years	1 748 000 (19.9)	633 (14.9)	58/633	9.16 (6.96-11.85)	5/633	0.79 (0.26-1.84)	19/633	3.00 (1.81-4.69)	239/633	37.76 (33.12-42.86)	57/633	9.00 (6.82-11.67)	66/633	10.43 (8.06-13.27)
% Crude prevalence (95% CI)			514/2972	17.29 (15.83-18.86)	102/2972	3.43 (2.80-4.17)	175/2972	5.89 (5.05-6.83)	1718/2972	57.81 (55.10-60.61)	641/2972	21.57 (19.93-23.30)	405/2972	13.63 (12.33-15.02)
% Age-adjusted prevalence (95% CI)				14.87 (13.54-16.20)		2.63 (2.07-3.19)		5.40 (4.55-6.24)		53.64 (51.71-55.56)		18.32 (16.90-19.73)		13.23 (11.92-14.55)
**Total**	8 777 000 (100.0)	4254 (100.0)	659/4254	15.49 (14.33-16.72)	126/4254	2.96 (2.47-3.53)	218/4254	5.12 (4.47-5.85)	2283/4254	53.67 (51.49-55.91)	833/4254	19.58 (18.27-20.96)	513/4254	12.06 (11.04-13.15)
**% Crude prevalence (95% CI)**				**15.49 (14.33-16.72)**		**2.96 (2.47-3.53)**		**5.12 (4.47-5.85)**		**53.67 (51.49-55.91)**		**19.58 (18.27-20.96)**		**12.06 (11.04-13.15)**
**% Sex- and age-adjusted prevalence[†] (95% CI)**				**12.80 (11.72-13.88)**		**2.15 (1.71-2.60)**		**4.42 (3.76-5.08)**		**48.73 (47.05-50.40)**		**16.73 (15.51-17.94)**		**10.91 (9.89-11.93)**

Supplementary Table S3.10. Prevalence of each type of dermographism of all participants in Russia (n=6335)

Age group	Russia population ≥ 18 years in 2023[‡] N (%)	Sample in this study n (%)	Point prevalence						Lifetime prevalence					
			Physiological red dermographism		Simple urticarial dermographism		Symptomatic dermographism		Physiological red dermographism		Simple urticarial dermographism		Symptomatic dermographism	
			n/N	% (95% CI)	n/N	% (95% CI)	n/N	% (95% CI)	n/N	% (95% CI)	n/N	% (95% CI)	n/N	% (95% CI)
**Male**														
18-24 years	5 156 000 (4.5)	628 (9.9)	322/628	51.27 (45.83-57.19)	54/628	8.60 (6.46-11.22)	21/628	3.34 (2.07-5.11)	468/628	74.52 (67.92-81.59)	130/628	20.70 (17.30-24.58)	44/628	7.01 (5.09-9.41)
25-60 years	35 312 000 (30.6)	798 (12.6)	232/798	29.07 (25.45-33.06)	43/798	5.39 (4.98-8.69)	38/798	4.76 (3.37-6.54)	457/798	57.27 (52.14-62.77)	160/798	20.05 (17.06-23.41)	163/798	20.43 (17.41-23.81)
> 60 years	11 579 000 (10.0)	731 (11.5)	376/731	51.44 (46.37-56.91)	27/731	3.69 (2.43-5.37)	35/731	4.79 (3.34-6.66)	524/731	71.68 (65.68-78.09)	87/731	11.90 (9.53-14.68)	100/731	13.68 (11.13-16.64)
% Crude prevalence (95% CI)			930/2157	43.12 (40.39-45.98)	124/2157	5.75 (4.78-6.85)	94/2157	4.36 (3.52-5.33)	1449/2157	67.18 (63.76-70.73)	377/2157	17.48 (15.76-19.33)	307/2157	14.23 (12.68-15.92)
% Age-adjusted prevalence (95% CI)				40.74 (38.91-42.57)		5.33 (4.20-6.46)		4.63 (3.56-5.70)		62.19 (59.72-64.65)		18.30 (16.32-20.28)		17.60 (15.61-19.59)
**Female**														
18-24 years	4 958 000 (4.3)	1408 (22.2)	883/1408	62.71 (58.64-66.99)	118/1408	8.38 (6.94-10.04)	69/1408	4.90 (3.81-6.20)	1151/1408	81.75 (77.09-86.61)	317/1408	22.51 (20.10-25.13)	178/1408	12.64 (10.85-14.64)
25-60 years	37 539 000 (32.6)	1467 (23.2)	556/1467	37.90 (34.82-41.19)	97/1467	6.61 (5.36-8.07)	116/1467	7.91 (6.53-9.48)	980/1467	66.80 (62.69-71.12)	318/1467	21.68 (19.36-24.20)	272/1467	18.54 (16.40-20.88)
> 60 years	20 715 000 (18.0)	1303 (20.6)	678/1303	52.03 (48.19-56.10)	66/1303	5.07 (3.92-6.44)	76/1303	5.83 (4.60-7.30)	961/1303	73.75 (69.16-78.57)	172/1303	13.20 (11.30-15.33)	204/1303	15.66 (13.58-17.96)
% Crude prevalence (95% CI)			2117/4178	50.67 (48.53-52.88)	281/4178	6.73 (5.96-7.56)	261/4178	6.25 (5.51-7.05)	3092/4178	74.01 (71.42-76.66)	807/4178	19.32 (18.01-20.70)	654/4178	15.65 (14.48-16.90)
% Age-adjusted prevalence (95% CI)				44.48 (42.74-46.21)		6.24 (5.39-7.10)		6.99 (6.07-7.92)		70.25 (68.61-71.89)		18.97 (17.57-20.37)		17.13 (15.78-18.49)
**Total**	115 259 000 (100.0)	6335 (100.0)	3047/6335	48.10 (46.41-49.84)	405/6335	6.39 (5.79-7.05)	355/6335	5.60 (5.04-6.22)	4541/6335	71.68 (69.61-73.80)	1184/6335	18.69 (17.64-19.79)	961/6335	15.17 (14.23-16.16)
**% Crude prevalence (95% CI)**				**48.10 (46.41-49.84)**		**6.39 (5.79-7.05)**		**5.60 (5.04-6.22)**		**71.68 (69.61-73.80)**		**18.69 (17.64-19.79)**		**15.17 (14.23-16.16)**
**% Sex- and age-adjusted prevalence[†] (95% CI)**				**40.76 (39.35-42.17)**		**5.83 (5.14-6.52)**		**5.92 (5.22-6.62)**		**66.61 (65.18-68.04)**		**18.67 (17.49-19.85)**		**17.34 (16.18-18.51)**

Supplementary Table S3.11. Prevalence of each type of dermographism of all participants in Saudi Arabia (n=3046)

Age group	Saudi Arabia population ≥ 18 years in 2023‡ N (%)	Sample in this study n (%)	Point prevalence						Lifetime prevalence					
			Physiological red dermographism		Simple urticarial dermographism		Symptomatic dermographism		Physiological red dermographism		Simple urticarial dermographism		Symptomatic dermographism	
			n/N	% (95% CI)	n/N	% (95% CI)	n/N	% (95% CI)	n/N	% (95% CI)	n/N	% (95% CI)	n/N	% (95% CI)
**Male**														
18-24 years	1 934 000 (8.1)	449 (14.7)	34/449	7.57 (5.24-10.58)	3/449	0.67 (0.14-1.95)	15/449	3.34 (1.87-5.51)	74/449	16.48 (12.94-20.69)	13/449	2.90 (1.54-4.95)	34/449	7.57 (5.24-10.58)
25-60 years	12 566 000 (52.8)	404 (13.3)	39/404	9.65 (6.87-13.20)	1/404	0.25 (0.00-1.38)	20/404	4.95 (3.02-7.65)	65/404	16.09 (12.42-20.51)	8/404	1.98 (0.86-3.90)	27/404	6.68 (4.40-9.72)
> 60 years	856 000 (3.6)	440 (14.4)	29/440	6.59 (4.41-9.47)	3/440	0.68 (0.14-1.99)	11/440	2.50 (1.25-4.47)	78/440	17.73 (14.01-22.12)	8/440	1.82 (0.79-3.58)	14/440	3.18 (1.74-5.34)
% Crude prevalence (95% CI)			102/1293	7.89 (6.43-9.58)	7/1293	0.54 (0.22-1.12)	46/1293	3.56 (2.61-4.75)	217/1293	16.78 (14.62-19.17)	29/1293	2.24 (1.50-3.22)	75/1293	5.80 (4.56-7.27)
% Age-adjusted prevalence (95% CI)				9.22 (6.84-11.60)		0.33 (0.09-0.74)		4.61 (2.87-6.36)		16.23 (13.26-19.20)		2.09 (0.96-3.22)		6.60 (4.58-8.62)
**Female**														
18-24 years	1 551 000 (6.5)	559 (18.4)	35/559	6.26 (4.36-8.71)	1/559	0.18 (0.00-1.00)	10/559	1.79 (0.86-3.29)	91/559	16.28 (13.11-19.99)	17/559	3.04 (1.77-4.87)	28/559	5.01 (3.33-7.24)
25-60 years	6 271 000 (26.3)	599 (19.7)	78/599	13.02 (10.29-16.25)	3/599	0.50 (0.10-1.46)	42/599	7.01 (5.05-9.48)	122/599	20.37 (16.91-24.32)	8/599	1.34 (0.58-2.63)	55/599	9.18 (6.92-11.95)
> 60 years	631 000 (2.6)	595 (19.5)	38/595	6.39 (4.52-8.77)	2/595	0.34 (0.04-1.21)	26/595	4.37 (2.85-6.40)	99/595	16.64 (13.52-20.26)	6/595	1.01 (0.37-2.20)	38/595	6.39 (4.52-8.77)
% Crude prevalence (95% CI)			151/1753	8.61 (7.30-10.10)	6/1753	0.34 (0.13-0.75)	78/1753	4.45 (3.52-5.55)	312/1753	17.80 (15.88-19.89)	31/1753	1.77 (1.20-2.51)	121/1753	6.90 (5.73-8.25)
% Age-adjusted prevalence (95% CI)				11.29 (9.25-13.32)		0.43 (0.00-0.86)		5.86 (4.32-7.39)		19.34 (16.87-21.81)		1.63 (0.89-2.36)		8.21 (6.45-9.96)
**Total**	23 809 000 (100.0)	3046 (100.0)	253/3046	8.31 (7.31-9.40)	13/3046	0.43 (0.23-0.73)	124/3046	4.07 (3.39-4.85)	529/3046	17.37 (15.92-18.91)	60/3046	1.97 (1.50-2.54)	196/3046	6.43 (5.57-7.40)
**% Crude prevalence (95% CI)**				**8.31 (7.31-9.40)**		**0.43 (0.23-0.73)**		**4.07 (3.39-4.85)**		**17.37 (15.92-18.91)**		**1.97 (1.50-2.54)**		**6.43 (5.57-7.40)**
**% Sex- and age-adjusted prevalence† (95% CI)**				**9.95 (8.26-11.65)**		**0.36 (0.06-0.67)**		**5.05 (3.80-6.30)**		**17.34 (15.23-19.44)**		**1.92 (1.15-2.70)**		**7.17 (5.73-8.61)**

Supplementary Table S3.12. Prevalence of each type of dermographism of all participants in Thailand (n=3247)

Age group	Thailand population ≥ 18 years in 2023[‡] N (%)	Sample in this study n (%)	Point prevalence						Lifetime prevalence					
			Physiological red dermographism		Simple urticarial dermographism		Symptomatic dermographism		Physiological red dermographism		Simple urticarial dermographism		Symptomatic dermographism	
			n/N	% (95% CI)	n/N	% (95% CI)	n/N	% (95% CI)	n/N	% (95% CI)	n/N	% (95% CI)	n/N	% (95% CI)
**Male**														
18-24 years	3 313 000 (5.7)	311 (9.6)	204/311	65.59 (56.90-75.24)	13/311	4.18 (2.23-7.15)	16/311	5.14 (2.94-8.36)	255/311	81.99 (72.24-92.70)	31/311	9.97 (6.77-14.15)	46/311	14.79 (10.83-19.73)
25-60 years	18 457 000 (31.6)	322 (9.9)	92/322	28.57 (23.03-35.04)	10/322	3.11 (1.49-5.71)	38/322	11.8 (8.35-16.20)	227/322	70.50 (61.62-80.29)	56/322	17.39 (13.14-22.58)	96/322	29.81 (24.15-36.41)
> 60 years	6 295 000 (10.8)	314 (9.7)	71/314	22.61 (17.66-28.52)	12/314	3.82 (1.96-6.68)	34/314	10.83 (7.50-15.13)	177/314	56.37 (48.37-65.31)	41/314	13.06 (9.37-17.71)	67/314	21.34 (16.54-27.10)
% Crude prevalence (95% CI)			367/947	38.75 (34.90-42.93)	35/947	3.70 (2.57-5.14)	88/947	9.29 (7.45-11.45)	659/947	69.59 (64.38-75.11)	128/947	13.52 (11.28-16.07)	209/947	22.07 (19.18-25.27)
% Age-adjusted prevalence (95% CI)				31.60 (28.14-35.07)		3.40 (2.04-4.76)		10.81 (8.35-13.27)		68.69 (65.15-72.22)		15.51 (12.64-18.39)		26.14 (22.67-29.61)
**Female**														
18-24 years	3 147 000 (5.4)	735 (22.6)	476/735	64.76 (46.44-55.69)	52/735	7.07 (5.28-9.28)	62/735	8.44 (6.47-10.81)	621/735	84.49 (77.97-91.40)	106/735	14.42 (11.81-17.44)	138/735	18.78 (15.77-22.18)
25-60 years	19 198 000 (32.9)	763 (23.5)	288/763	37.75 (33.51-42.37)	63/763	8.26 (6.35-10.56)	108/763	14.15 (11.61-17.09)	550/763	72.08 (66.18-78.37)	166/763	21.76 (18.57-25.33)	224/763	29.36 (25.64-33.46)
> 60 years	7 910 000 (13.6)	802 (24.7)	213/802	26.56 (23.11-30.37)	33/802	4.11 (2.83-5.78)	82/802	10.22 (8.13-12.69)	489/802	60.97 (55.69-66.62)	127/802	15.84 (13.20-18.84)	202/802	25.19 (21.83-28.91)
% Crude prevalence (95% CI)			977/2300	42.48 (39.86-45.23)	148/2300	6.43 (5.44-7.56)	252/2300	10.96 (9.63-12.40)	1660/2300	72.17 (68.74-75.73)	399/2300	17.35 (15.69-19.14)	564/2300	24.52 (22.54-26.63)
% Age-adjusted prevalence (95% CI)				37.63 (35.28-39.99)		7.05 (5.76-8.36)		12.53 (10.85-14.20)		70.47 (68.25-72.69)		19.45 (17.46-21.44)		27.17 (24.95-29.39)
**Total**	58 320 000 (100.0)	3247 (100.0)	1344/3247	41.39 (39.21-43.67)	183/3247	5.64 (4.85-6.51)	340/3247	10.47 (9.39-11.65)	2319/3247	71.42 (68.54-74.39)	527/3247	16.23 (14.87-17.68)	773/3247	23.81 (22.16-25.55)
**% Crude prevalence (95% CI)**				**41.39 (39.21-43.67)**		**5.64 (4.85-6.51)**		**10.47 (9.39-11.65)**		**71.42 (68.54-74.39)**		**16.23 (14.87-17.68)**		**23.81 (22.16-25.55)**
**% Sex- and age-adjusted prevalence[†] (95% CI)**				**34.73 (32.66-36.80)**		**5.29 (4.35-6.23)**		**11.70 (10.23-13.17)**		**69.61 (67.56-71.67)**		**17.55 (15.83-19.28)**		**26.67 (24.65-28.70)**

Supplementary Table S3.13. Prevalence of each type of dermographism of all participants in Brazil (n=1584)

Age group	Brazil population ≥ 18 years in 2023[‡] N (%)	Sample in this study n (%)	Point prevalence						Lifetime prevalence					
			Physiological red dermographism		Simple urticarial dermographism		Symptomatic dermographism		Physiological red dermographism		Simple urticarial dermographism		Symptomatic dermographism	
			n/N	% (95% CI)	n/N	% (95% CI)	n/N	% (95% CI)	n/N	% (95% CI)	n/N	% (95% CI)	n/N	% (95% CI)
**Male**														
18-24 years	11 235 000 (7.0)	79 (5.0)	23/79	29.11 (18.46-43.69)	0/79	0.00 N/A	5/79	6.33 (2.06-14.77)	35/79	44.30 (30.86-61.62)	5/79	6.33 (2.06-14.77)	5/79	6.33 (2.06-14.77)
25-60 years	52 893 000 (33.0)	258 (16.3)	78/258	30.23 (23.90-37.73)	10/258	3.88 (1.86-7.13)	22/258	8.53 (5.34-12.91)	119/258	46.12 (38.21-55.19)	29/258	11.24 (7.53-16.14)	28/258	10.85 (7.21-15.69)
> 60 years	13 754 000 (8.6)	68 (4.3)	8/68	11.76 (5.08-23.18)	0/68	0.00 N/A	1/68	1.47 (0.04-8.19)	18/68	26.47 (15.69-41.83)	4/68	5.88 (1.60-15.06)	2/68	2.94 (0.36-10.63)
% Crude prevalence (95% CI)			109/405	26.91 (22.10-32.47)	10/405	2.47 (1.18-4.54)	28/405	6.91 (4.59-9.99)	172/405	42.47 (36.36-49.31)	38/405	9.38 (6.64-12.88)	35/405	8.64 (6.02-12.02)
% Age-adjusted prevalence (95% CI)				26.81 (22.52-31.10)		3.88 (1.52-6.24)		6.97 (4.47-9.46)		42.39 (37.59-47.18)		9.59 (6.68-12.49)		8.80 (6.02-11.58)
**Female**														
18-24 years	10 922 000 (6.8)	209 (13.2)	70/209	33.49 (26.11-42.32)	8/209	3.83 (1.65-7.54)	26/209	12.44 (8.13-18.23)	110/209	52.63 (43.26-63.44)	35/209	16.75 (11.66-23.29)	33/209	15.79 (10.87-22.17)
25-60 years	54 358 000 (34.0)	831 (52.5)	280/831	33.69 (29.86-37.88)	32/831	3.85 (2.63-5.44)	90/831	10.83 (8.71-13.31)	491/831	59.09 (53.97-64.55)	128/831	15.4 (12.85-18.31)	142/831	17.09 (14.39-20.14)
> 60 years	16 935 000 (10.6)	139 (8.8)	20/139	14.39 (8.79-22.22)	1/139	0.72 (0.02-4.01)	5/139	3.60 (1.17-8.39)	49/139	35.25 (26.08-46.60)	13/139	9.35 (4.98-15.99)	11/139	7.91 (3.95-14.16)
% Crude prevalence (95% CI)			370/1179	31.38 (28.27-34.75)	41/1179	3.48 (2.50-4.72)	121/1179	10.26 (8.52-12.26)	650/1179	55.13 (50.97-59.54)	176/1179	14.93 (12.80-17.30)	186/1179	15.78 (13.59-18.21)
% Age-adjusted prevalence (95% CI)				29.69 (27.10-32.27)		3.20 (2.23-4.18)		9.56 (7.91-11.20)		53.32 (50.43-56.21)		14.33 (12.31-16.35)		15.03 (12.99-17.06)
**Total**	160 097 000 (100.0)	1584 (100.0)	479/1584	30.24 (27.59-33.07)	51/1584	3.22 (2.40-4.23)	149/1584	9.41 (7.96-11.04)	822/1584	51.89 (48.41-55.57)	214/1584	13.51 (11.76-15.45)	221/1584	13.95 (12.17-15.92)
**% Crude prevalence (95% CI)**				**30.24 (27.59-33.07)**		**3.22 (2.40-4.23)**		**9.41 (7.96-11.04)**		**51.89 (48.41-55.57)**		**13.51 (11.76-15.45)**		**13.95 (12.17-15.92)**
**% Sex- and age-adjusted prevalence[†] (95% CI)**				**28.29 (25.81-30.76)**		**3.47 (2.37-4.57)**		**8.30 (6.82-9.77)**		**48.00 (45.24-50.77)**		**12.02 (10.27-13.78)**		**12.00 (10.29-13.71)**

Supplementary Table S3.14. Prevalence of each type of dermographism of all participants in Georgia (n=1576)

Age group	Georgia population ≥ 18 years in 2023[‡] N (%)	Sample in this study n (%)	Point prevalence						Lifetime prevalence					
			Physiological red dermographism		Simple urticarial dermographism		Symptomatic dermographism		Physiological red dermographism		Simple urticarial dermographism		Symptomatic dermographism	
			n/N	% (95% CI)	n/N	% (95% CI)	n/N	% (95% CI)	n/N	% (95% CI)	n/N	% (95% CI)	n/N	% (95% CI)
**Male**														
18-24 years	153 000 (5.3)	238 (15.1)	16/238	6.72 (3.84-10.92)	4/238	1.68 (0.46-4.30)	3/238	1.26 (0.26-3.68)	98/238	41.18 (33.43-50.18)	14/238	5.88 (3.22-9.87)	9/238	3.78 (1.73-7.18)
25-60 years	851 000 (29.7)	351 (22.3)	15/351	4.27 (2.39-7.05)	0/351	0.00 N/A	2/351	0.57 (0.07-2.06)	96/351	27.35 (22.15-33.40)	6/351	1.71 (0.63-3.72)	9/351	2.56 (1.17-4.87)
> 60 years	287 000 (10.0)	87 (5.5)	5/87	5.75 (1.87-13.41)	0/87	0.00 N/A	1/87	1.15 (0.03-6.40)	24/87	27.59 (17.68-41.05)	3/87	3.45 (0.71-10.08)	4/87	4.60 (1.25-11.77)
% Crude prevalence (95% CI)			36/676	5.33 (3.73-7.37)	4/676	0.59 (0.16-1.52)	6/676	0.89 (0.33-1.93)	218/676	32.25 (28.11-36.83)	23/676	3.40 (2.16-5.11)	22/676	3.25 (1.04-4.93)
% Age-adjusted prevalence (95% CI)				4.89 (3.08-6.70)		1.68 (0.05-3.31)		0.78 (0.04-1.52)		29.04 (25.25-32.83)		2.59 (1.31-3.88)		3.16 (1.67-4.65)
**Female**														
18-24 years	144 000 (5.0)	348 (22.1)	42/348	12.07 (8.70-16.31)	9/348	2.59 (1.18-4.91)	8/348	2.30 (0.99-4.53)	150/348	43.10 (36.48-50.58)	40/348	11.49 (8.21-15.65)	17/348	4.89 (2.85-7.82)
25-60 years	944 000 (32.9)	416 (26.4)	42/416	10.1 (7.28-13.65)	5/416	1.2 (0.39-2.81)	5/416	1.20 (0.39-2.81)	143/416	34.38 (28.97-40.49)	17/416	4.09 (2.38-6.54)	18/416	4.33 (2.56-6.84)
> 60 years	490 000 (17.1)	136 (8.6)	4/136	2.94 (0.80-7.53)	0/136	0.00 N/A	1/136	0.74 (0.02-4.10)	30/136	22.06 (14.88-31.49)	2/136	1.47 (0.18-5.31)	4/136	2.94 (0.80-7.53)
% Crude prevalence (95% CI)			88/900	9.78 (7.84-12.05)	14/900	1.56 (0.85-2.61)	14/900	1.56 (0.85-2.61)	323/900	35.89 (32.08-40.02)	59/900	6.56 (4.99-8.46)	39/900	4.33 (3.08-5.92)
% Age-adjusted prevalence (95% CI)				8.06 (6.09-10.03)		1.38 (0.45-2.32)		1.16 (0.38-1.94)		31.35 (27.84-34.87)		3.95 (2.62-5.29)		3.95 (2.47-5.43)
**Total**	2 869 000 (100.0)	1576 (100.0)	124/1576	7.87 (6.54-9.38)	18/1576	1.14 (0.68-1.81)	20/1576	1.27 (0.78-1.96)	541/1576	34.33 (31.50-37.35)	82/1576	5.20 (4.14-6.46)	61/1576	3.87 (2.96-4.97)
**% Crude prevalence (95% CI)**				**7.87 (6.54-9.38)**		**1.14 (0.68-1.81)**		**1.27 (0.78-1.96)**		**34.33 (31.50-37.35)**		**5.20 (4.14-6.46)**		**3.87 (2.96-4.97)**
**% Sex- and age-adjusted prevalence[†] (95% CI)**				**6.63 (5.28-7.99)**		**1.42 (0.58-2.26)**		**0.99 (0.44-1.53)**		**30.31 (27.73-32.89)**		**3.34 (2.41-4.27)**		**3.59 (2.54-4.65)**

Supplementary Table S3.15. Prevalence of each type of dermographism of all participants in Japan (n=2068)

Age group	Japan population ≥ 18 years in 2023[‡] N (%)	Sample in this study n (%)	Point prevalence						Lifetime prevalence					
			Physiological red dermographism		Simple urticarial dermographism		Symptomatic dermographism		Physiological red dermographism		Simple urticarial dermographism		Symptomatic dermographism	
			n/N	% (95% CI)	n/N	% (95% CI)	n/N	% (95% CI)	n/N	% (95% CI)	n/N	% (95% CI)	n/N	% (95% CI)
**Male**														
18-24 years	4 296 000 (4.0)	87 (4.2)	21/87	24.14 (14.94-36.90)	2/87	2.30 (0.28-8.30)	3/87	3.45 (0.71-10.08)	49/87	56.32 (41.67-74.46)	18/87	20.69 (12.26-32.70)	7/87	8.05 (3.24-16.58)
25-60 years	28 050 000 (26.3)	402 (19.4)	87/402	21.64 (17.33-26.70)	13/402	3.23 (1.72-5.53)	24/402	5.97 (3.83-8.88)	229/402	56.97 (49.83-64.84)	71/402	17.66 (13.79-22.28)	63/402	15.67 (12.04-20.05)
> 60 years	19 181 000 (18.0)	104 (5.0)	12/104	11.54 (5.96-20.16)	1/104	0.96 (0.02-5.36)	2/104	1.92 (0.23-6.95)	57/104	54.81 (41.51-71.01)	31/104	29.81 (20.25-42.31)	10/104	9.62 (4.61-17.68)
% Crude prevalence (95% CI)			120/593	20.24 (16.78-24.20)	16/593	2.70 (1.54-4.38)	29/593	4.89 (3.28-7.02)	335/593	56.49 (50.60-62.88)	120/593	20.24 (16.78-24.20)	80/593	13.49 (10.70-16.79)
% Age-adjusted prevalence (95% CI)				18.09 (14.84-21.34)		2.31 (1.11-3.51)		4.25 (2.62-5.88)		56.11 (51.60-60.63)		22.44 (18.52-26.35)		12.78 (9.88-15.68)
**Female**														
18-24 years	4 078 000 (3.8)	244 (11.8)	54/244	22.13 (16.63-28.88)	12/244	4.92 (2.54-8.59)	9/244	3.69 (1.69-7.00)	158/244	64.75 (55.05-75.68)	81/244	33.20 (26.36-41.26)	36/244	14.75 (10.33-20.43)
25-60 years	27 320 000 (25.6)	1080 (52.2)	331/1080	30.65 (27.43-34.13)	47/1080	4.35 (3.20-5.79)	136/1080	12.59 (10.57-14.90)	824/1080	76.30 (71.18-81.69)	270/1080	25.00 (22.11-28.17)	354/1080	32.78 (29.45-36.38)
> 60 years	23 589 000 (22.1)	151 (7.3)	24/151	15.89 (10.18-23.65)	6/151	3.97 (1.46-8.65)	14/151	9.27 (5.07-15.56)	93/151	61.59 (49.71-75.45)	39/151	25.83 (18.37-35.31)	34/151	22.52 (15.59-31.46)
% Crude prevalence (95% CI)			409/1475	27.73 (25.11-30.55)	65/1475	4.41 (3.40-5.62)	159/1475	10.78 (9.17-12.59)	1075/1475	72.88 (68.59-77.37)	390/1475	26.44 (23.88-29.20)	424/1475	28.75 (26.07-31.62)
% Age-adjusted prevalence (95% CI)				23.69 (20.81-26.56)		4.23 (2.75-5.71)		10.51 (8.28-12.73)		69.13 (65.55-72.72)		25.96 (22.68-29.25)		27.04 (23.85-30.24)
**Total**	106 514 000 (100.0)	2068 (100.0)	529/2068	25.58 (23.45-27.86)	81/2068	3.92 (3.11-4.87)	188/2068	9.09 (7.84-10.49)	1410/2068	68.18 (64.67-71.84)	510/2068	24.66 (22.57-26.90)	504/2068	24.37 (22.29-26.59)
**% Crude prevalence (95% CI)**				25.58 (23.45-27.86)		3.92 (3.11-4.87)		9.09 (7.84-10.49)		68.18 (64.67-71.84)		24.66 (22.57-26.90)		24.37 (22.29-26.59)
**% Sex- and age-adjusted prevalence[†] (95% CI)**				**20.98 (18.81-23.14)**		**3.30 (2.34-4.26)**		**7.48 (6.09-8.87)**		**62.83 (59.97-65.70)**		**24.26 (21.71-26.80)**		**20.14 (17.98-22.31)**

Supplementary Table S3.16. Prevalence of each type of dermographism of all participants in North Macedonia (n=1287)

Age group	North Macedonia population ≥ 18 years in 2023[‡] N (%)	Sample in this study n (%)	Point prevalence						Lifetime prevalence					
			Physiological red dermographism		Simple urticarial dermographism		Symptomatic dermographism		Physiological red dermographism		Simple urticarial dermographism		Symptomatic dermographism	
			n/N	% (95% CI)	n/N	% (95% CI)	n/N	% (95% CI)	n/N	% (95% CI)	n/N	% (95% CI)	n/N	% (95% CI)
**Male**														
18-24 years	75 000 (5.1)	53 (4.1)	18/53	33.96 (20.13-53.68)	1/53	1.89 (0.05-10.51)	6/53	11.32 (4.15-24.64)	34/53	64.15 (44.43-89.64)	10/53	18.87 (9.05-34.70)	8/53	15.09 (6.52-29.74)
25-60 years	441 000 (30.3)	158 (12.3)	40/158	25.32 (18.09-34.47)	4/158	2.53 (0.69-6.48)	14/158	8.86 (4.84-14.87)	91/158	57.59 (46.37-70.71)	21/158	13.29 (8.23-20.32)	25/158	15.82 (10.24-23.36)
> 60 years	184 000 (12.6)	82 (6.4)	12/82	14.63 (7.56-25.56)	3/82	3.66 (0.75-10.69)	4/82	4.88 (1.33-12.49)	34/82	41.46 (28.71-57.94)	8/82	9.76 (4.21-19.22)	8/82	9.76 (4.21-19.22)
% Crude prevalence (95% CI)			70/293	23.89 (18.62-30.18)	8/293	2.73 (1.18-5.38)	24/293	8.19 (5.25-12.19)	159/293	54.27 (46.16-63.39)	39/293	13.31 (9.47-18.20)	41/293	13.99 (10.04-18.98)
% Age-adjusted prevalence (95% CI)				23.44 (18.52-28.35)		2.76 (0.84-4.68)		8.08 (4.90-11.26)		54.05 (48.28-59.83)		12.96 (9.06-16.86)		14.15 (10.06-18.24)
**Female**														
18-24 years	73 000 (5.0)	196 (15.2)	28/196	14.29 (9.49-20.65)	10/196	5.10 (2.45-9.38)	8/196	4.08 (1.76-8.04)	157/196	80.10 (68.06-93.66)	64/196	32.65 (25.15-41.70)	27/196	13.78 (9.08-20.04)
25-60 years	450 000 (30.9)	676 (52.5)	166/676	24.56 (20.96-28.59)	27/676	3.99 (2.63-5.81)	63/676	9.32 (7.16-11.92)	464/676	68.64 (62.53-75.18)	177/676	26.18 (22.47-30.34)	117/676	17.31 (14.31-20.74)
> 60 years	234 000 (16.1)	122 (9.5)	19/122	15.57 (9.38-24.32)	6/122	4.92 (1.81-10.70)	3/122	2.46 (0.51-7.19)	53/122	43.44 (32.54-56.82)	13/122	10.66 (5.67-18.22)	18/122	14.75 (8.74-23.32)
% Crude prevalence (95% CI)			213/994	21.43 (18.65-24.51)	43/994	4.33 (3.13-5.83)	74/994	7.44 (5.85-9.35)	674/994	67.81 (62.78-73.12)	254/994	25.55 (22.51-28.90)	162/994	16.30 (13.88-19.01)
% Age-adjusted prevalence (95% CI)				20.79 (17.98-23.60)		4.39 (2.88-5.89)		6.69 (5.12-8.27)		61.96 (58.49-65.42)		22.01 (19.34-24.68)		16.18 (13.56-18.80)
**Total**	1 458 000 (100.0)	1287 (100.0)	283/1287	21.99 (19.50-24.71)	51/1287	3.96 (2.95-5.21)	98/1287	7.61 (6.18-9.28)	833/1287	64.72 (60.40-69.27)	293/1287	22.77 (20.23-25.53)	203/1287	15.77 (13.68-18.10)
**% Crude prevalence (95% CI)**				**21.99 (19.50-24.71)**		**3.96 (2.95-5.21)**		**7.61 (6.18-9.28)**		**64.72 (60.40-69.27)**		**22.77 (20.23-25.53)**		**15.77 (13.68-18.10)**
**% Sex- and age-adjusted prevalence[†] (95% CI)**				**22.06 (19.29-24.84)**		**3.60 (2.40-4.81)**		**7.36 (5.62-9.09)**		**58.16 (54.85-61.47)**		**17.66 (15.33-19.99)**		**15.20 (12.81-17.60)**

Supplementary Table S3.17. Prevalence of each type of dermographism of all participants in South Korea (n=3410)

Age group	South Korea population ≥ 18 years in 2023[‡] N (%)	Sample in this study n (%)	Point prevalence						Lifetime prevalence					
			Physiological red dermographism		Simple urticarial dermographism		Symptomatic dermographism		Physiological red dermographism		Simple urticarial dermographism		Symptomatic dermographism	
			n/N	% (95% CI)	n/N	% (95% CI)	n/N	% (95% CI)	n/N	% (95% CI)	n/N	% (95% CI)	n/N	% (95% CI)
**Male**														
18-24 years	2 042 000 (4.6)	329 (9.6)	96/329	29.18 (23.64-35.63)	13/329	3.95 (2.10-6.76)	23/329	6.99 (4.43-10.49)	133/329	40.43 (33.85-47.91)	20/329	6.08 (3.71-9.39)	40/329	12.16 (8.69-16.56)
25-60 years	14 369 000 (32.1)	1052 (30.9)	362/1052	34.41 (30.96-38.14)	104/1052	9.89 (8.08-11.98)	87/1052	8.27 (6.62-10.20)	509/1052	48.38 (44.27-52.78)	180/1052	17.11 (14.70-19.80)	134/1052	12.74 (10.67-15.09)
> 60 years	5 801 000 (13.0)	265 (7.8)	50/265	18.87 (14.00-24.88)	3/265	1.13 (0.23-3.31)	12/265	4.53 (2.34-7.91)	103/265	38.87 (31.73-47.14)	7/265	2.64 (1.06-5.44)	28/265	10.57 (7.02-15.27)
% Crude prevalence (95% CI)			508/1646	30.86 (28.24-33.67)	120/1646	7.29 (6.04-8.72)	122/1646	7.41 (6.16-8.85)	745/1646	45.26 (42.07-48.63)	207/1646	12.58 (10.92-14.41)	202/1646	12.27 (10.64-14.09)
% Age-adjusted prevalence (95% CI)				29.87 (27.60-32.14)		7.06 (5.83-8.29)		7.18 (5.89-8.46)		45.17 (42.64-47.70)		12.32 (10.74-13.89)		12.12 (10.47-13.78)
**Female**														
18-24 years	1 876 000 (4.2)	411 (12.1)	141/411	34.31 (28.88-40.46)	30/411	7.30 (4.93-10.42)	33/411	8.03 (5.53-11.28)	185/411	45.01 (38.76-51.99)	51/411	12.41 (9.24-16.32)	50/411	12.17 (9.03-16.04)
25-60 years	13 540 000 (30.3)	981 (28.8)	418/981	42.61 (38.62-46.90)	82/981	8.36 (6.65-10.38)	150/981	15.29 (12.94-17.94)	572/981	58.31 (53.63-63.29)	154/981	15.70 (13.32-18.38)	239/981	24.36 (21.37-27.66)
> 60 years	7 076 000 (15.8)	372 (10.9)	71/372	19.09 (14.91-24.07)	2/372	0.54 (0.07-1.94)	15/372	4.03 (2.26-6.65)	154/372	41.40 (35.12-48.48)	8/372	2.15 (0.93-4.24)	42/372	11.29 (8.14-15.26)
% Crude prevalence (95% CI)			630/1764	35.71 (32.98-38.62)	114/1764	6.46 (5.33-7.76)	198/1764	11.22 (9.72-12.90)	911/1764	51.64 (48.34-55.11)	213/1764	12.07 (10.51-13.81)	331/1764	18.76 (16.80-20.90)
% Age-adjusted prevalence (95% CI)				34.52 (32.24-36.80)		5.81 (4.72-6.90)		11.14 (9.63-12.65)		51.88 (49.41-54.35)		11.16 (9.69-12.63)		19.23 (17.31-21.16)
**Total**	44 704 000 (100.0)	3410 (100.0)	1138/3410	33.37 (31.46-35.37)	234/3410	6.86 (6.01-7.80)	320/3410	9.38 (8.38-10.47)	1656/3410	48.56 (46.25-50.96)	420/3410	12.32 (11.17-13.55)	533/3410	15.63 (14.33-17.02)
**% Crude prevalence (95% CI)**				33.37 (31.46-35.37)		6.86 (6.01-7.80)		9.38 (8.38-10.47)		48.56 (46.25-50.96)		12.32 (11.17-13.55)		15.63 (14.33-17.02)
**% Sex- and age-adjusted prevalence[†] (95% CI)**				**32.21 (30.60-33.82)**		**6.43 (5.61-7.25)**		**9.17 (8.18-10.16)**		**48.54 (46.78-50.31)**		**11.74 (10.66-12.81)**		**15.70 (14.43-16.97)**

Supplementary Table S3.18. Prevalence of each type of dermographism of all participants in Spain (n=525)

Age group	Spain population ≥ 18 years in 2023‡ N (%)	Sample in this study n (%)	Point prevalence						Lifetime prevalence					
			Physiological red dermographism		Simple urticarial dermographism		Symptomatic dermographism		Physiological red dermographism		Simple urticarial dermographism		Symptomatic dermographism	
			n/N	% (95% CI)	n/N	% (95% CI)	n/N	% (95% CI)	n/N	% (95% CI)	n/N	% (95% CI)	n/N	% (95% CI)
**Male**														
18-24 years	1 767 000 (4.4)	16 (3.0)	5/16	31.25 (10.15-72.93)	1/16	6.25 (0.16-34.82)	0/16	0.00 N/A	12/16	75.00 (38.75-100.00)	4/16	25.00 (6.81-64.01)	0/16	0.00 N/A
25-60 years	12 148 000 (30.3)	115 (21.9)	27/115	23.48 (15.47-34.16)	4/115	3.48 (0.95-8.91)	3/115	2.61 (0.54-7.62)	68/115	59.13 (45.92-74.96)	27/115	23.48 (15.47-34.16)	5/115	4.35 (1.41-10.15)
> 60 years	5 553 000 (13.9)	42 (8.0)	2/42	4.76 (0.58-17.20)	0/42	0.00 N/A	0/42	0.00 N/A	11/42	26.19 (13.07-46.86)	3/42	7.14 (1.47-20.87)	1/42	2.38 (0.06-13.27)
% Crude prevalence (95% CI)			34/173	19.65 (13.61-27.46)	5/173	2.89 (0.94-6.75)	3/173	1.73 (0.36-5.07)	91/173	52.60 (42.35-64.58)	34/173	19.65 (13.61-27.46)	6/173	3.47 (1.27-7.55)
% Age-adjusted prevalence (95% CI)				18.85 (13.28-24.41)		3.83 (0.54-7.12)		2.61 (0-5.52)		51.18 (44.14-58.21)		18.96 (13.30-24.62)		3.73 (0.79-6.67)
**Female**														
18-24 years	1 664 000 (4.2)	19 (3.6)	10/19	52.63 (25.24-96.79)	2/19	10.53 (1.27-38.02)	1/19	5.26 (0.13-29.32)	15/19	78.95 (44.19-100.00)	5/19	26.32 (8.54-61.41)	1/19	5.26 (0.13-29.32)
25-60 years	11 997 000 (30.0)	245 (46.7)	81/245	33.06 (26.26-41.09)	17/245	6.94 (4.04-11.11)	23/245	9.39 (5.95-14.09)	169/245	68.98 (58.97-80.20)	53/245	21.63 (16.2-18.3)	37/245	15.10 (10.63-20.82)
> 60 years	6 901 000 (17.2)	88 (16.8)	26/88	29.55 (19.30-43.29)	2/88	2.27 (0.28-8.21)	3/88	3.41 (0.70-9.96)	50/88	56.82 (42.17-74.91)	13/88	14.77 (7.87-25.26)	17/88	19.32 (11.25-30.93)
% Crude prevalence (95% CI)			117/352	33.24 (27.49-39.84)	21/352	5.97 (3.69-9.12)	27/352	7.67 (5.06-11.16)	234/352	66.48 (58.23-75.56)	71/352	20.17 (15.75-25.44)	55/352	15.63 (11.77-20.34)
% Age-adjusted prevalence (95% CI)				33.47 (28.43-38.50)		5.66 (3.26-8.07)		7.05 (4.44-9.66)		65.71 (60.64-70.77)		19.71 (15.49-23.93)		15.72 (11.83-19.61)
**Total**	40 030 000 (100.0)	525 (100.0)	151/525	28.76 (24.36-33.73)	26/525	4.95 (3.24-7.26)	30/525	5.71 (3.86-8.16)	325/525	61.90 (55.36-69.01)	105/525	20.00 (16.36-24.21)	61/525	11.62 (8.89-14.93)
**% Crude prevalence (95% CI)**				28.76 (24.36-33.73)		4.95 (3.24-7.26)		5.71 (3.86-8.16)		61.90 (55.36-69.01)		20.00 (16.36-24.21)		11.62 (8.89-14.93)
**% Sex- and age-adjusted prevalence† (95% CI)**				**26.35 (22.61-30.10)**		**4.92 (2.97-6.88)**		**5.40 (3.43-7.37)**		**58.64 (54.34-62.94)**		**19.34 (15.84-22.85)**		**10.17 (7.68-12.67)**

Supplementary Table S3.19. Prevalence of each type of dermographism of all participants in Türkiye (n =2265)

Age group	Türkiye population ≥ 18 years in 2023‡ N (%)	Sample in this study n (%)	Point prevalence						Lifetime prevalence					
			Physiological red dermographism		Simple urticarial dermographism		Symptomatic dermographism		Physiological red dermographism		Simple urticarial dermographism		Symptomatic dermographism	
			n/N	% (95% CI)	n/N	% (95% CI)	n/N	% (95% CI)	n/N	% (95% CI)	n/N	% (95% CI)	n/N	% (95% CI)
**Male**														
18-24 years	4 758 000 (7.4)	249 (11)	58/249	23.29 (17.69-30.11)	20/249	8.03 (4.91-12.41)	3/249	1.20 (0.25-3.52)	128/249	51.41 (42.89-61.12)	52/249	20.88 (15.60-27.39)	7/249	2.81 (0.10-2.90)
25-60 years	21 863 000 (33.9)	432 (19.1)	108/432	25.00 (20.51-30.18)	22/432	5.09 (3.19-7.71)	9/432	2.08 (0.95-3.96)	211/432	48.84 (42.47-55.90)	77/432	17.82 (14.07-22.28)	24/432	5.56 (3.56-8.27)
> 60 years	5 320 000 (8.3)	211 (9.3)	45/211	21.33 (15.56-28.54)	4/211	1.90 (0.52-4.85)	1/211	0.47 (0.01-2.64)	79/211	37.44 (29.64-46.66)	11/211	5.21 (2.60-9.33)	11/211	5.21 (2.60-9.33)
% Crude prevalence (95% CI)			211/892	23.65 (20.57-27.07)	46/892	5.16 (3.78-6.88)	13/892	1.46 (0.78-2.49)	418/892	46.86 (42.48-51.58)	140/892	15.70 (13.20-18.52)	42/892	4.71 (3.39-6.37)
% Age-adjusted prevalence (95% CI)				24.13 (21.09-27.18)		5.00 (3.46-6.53)		1.68 (0.73-2.64)		47.31 (43.80-50.85)		16.18 (13.55-18.81)		5.09 (3.50-6.68)
**Female**														
18-24 years	4 560 000 (7.1)	466 (20.6)	184/466	39.48 (33.99-45.62)	59/466	12.66 (9.64-16.33)	16/466	3.43 (1.96-5.58)	323/466	69.31 (61.96-77.30)	136/466	29.18 (24.49-34.52)	35/466	7.51 (5.23-10.45)
25-60 years	21 500 000 (33.4)	623 (27.5)	220/623	35.31 (30.80-40.30)	50/623	8.03 (5.96-10.58)	39/623	6.26 (4.45-8.56)	389/623	62.44 (56.39-68.96)	133/623	21.35 (17.87-25.30)	80/623	12.84 (10.18-15.98)
> 60 years	6 427 000 (10.0)	284 (12.5)	80/284	28.17 (22.34-35.06)	7/284	2.46 (0.99-5.08)	12/284	4.23 (2.18-7.38)	132/284	46.48 (38.89-55.12)	24/284	8.45 (5.42-12.57)	19/284	6.69 (4.03-10.45)
% Crude prevalence (95% CI)			484/1373	35.25 (32.18-38.54)	116/1373	8.45 (6.98-10.13)	67/1373	4.88 (3.78-6.20)	844/1373	61.47 (57.39-65.76)	293/1373	21.34 (18.97-23.93)	134/1373	9.76 (8.18-11.56)
% Age-adjusted prevalence (95% CI)				34.68 (31.92-37.45)		7.58 (6.06-9.10)		5.46 (4.10-6.82)		60.25 (57.42-63.08)		19.90 (17.60-22.20)		10.88 (9.01-12.74)
**Total**	64 428 000 (100.0)	2265 (100.0)	695/2265	30.68 (28.45-33.05)	162/2265	7.15 (6.09-8.34)	80/2265	3.53 (2.80-4.40)	1262/2265	55.72 (52.69-58.88)	433/2265	19.12 (17.36-21.01)	176/2265	7.77 (6.67-9.01)
**% Crude prevalence (95% CI)**				**30.68 (28.45-33.05)**		**7.15 (6.09-8.34)**		**3.53 (2.80-4.40)**		**55.72 (52.69-58.88)**		**19.12 (17.36-21.01)**		**7.77 (6.67-9.01)**
**% Sex- and age-adjusted prevalence† (95% CI)**				**29.43 (27.40-31.51)**		**6.30 (5.22-7.38)**		**3.59 (2.75-4.42)**		**53.84 (51.58-56.10)**		**18.05 (16.31-19.80)**		**8.01 (6.78-9.23)**

† Prevalence is adjusted based on 6 strata by sex and age group distribution in each country's population
‡ Standard population was obtained from United Nations, World Population Prospects 2024
Abbreviations: NA, not applicable

Supplementary Table S4. Participant recruitment: channels to distribute the internet-based questionnaire (n=19)

Distribution channel†	n/Total (%)
**Social media**	18/19 (94.7)
WhatsApp	15/18 (83.3)
Instagram	9/18 (50.0)
Facebook	9/18 (50.0)
Email group	4/18 (22.2)
Telegram	3/18 (16.7)
X (Twitter)	2/18 (11.1)
Others	
Reddit	1/18 (5.6)
LinkedIn	1/18 (5.6)
LINE application	1/18 (5.6)
VKontakte	1/18 (5.6)
Odnoklassniki	1/18 (5.6)
KakaoTalk	1/18 (5.6)
**Random and anonymous direct invitations in a public place**	14/19 (73.7)
**Online forums or communities**	8/19 (42.1)

† Each center can have more than one distribution channel.

Supplementary Table S5. Number of participating responses

Country	Number of participating responses	Number of non-participating responses	Non-participation rate (%)
**Ecuador**	3582	87	2.371
**Germany**	3541	134	3.784
**India**	3092	28	0.897
**Iran**	3253	41	1.260
**Kuwait**	3556	2	0.056
**Oman**	7106	71	0.999
**Peru**	6447	239	3.707
**Poland**	12084	970	7.431
**Portugal**	4254	55	1.276
**Russia**	6335	62	0.969
**Saudi Arabia**	3046	191	5.901
**Thailand**	3247	343	9.554
**Brazil**	1584	5	0.315
**Georgia**	1576	25	1.562
**Japan**	2068	88	4.082
**North Macedonia**	1287	13	1.000
**South Korea**	3410	14	0.409
**Spain**	525	6	1.130
**Türkiye**	2265	64	2.748

Supplementary Table S6. Comparison of prevalence of symptomatic dermographism by age group across 12 countries

	All age		Age 18-24 years		Age 25-60 years		Age >60 years		*P* value	***P* value** between group		
	**Total**	**n (%)**	**Total**	**n (%)**	**Total**	**n (%)**	**Total**	**n (%)**		18-24 years and 25-60 years	18-24 years and >60 years	25-60 years and >60 years
**International**												
Point prevalence of SD	59 543	2216 (3.7)	18 140	572 (3.2)	24 343	1188 (4.9)	17 060	456 (2.7)	<0.001*	*	*	*
Male	23 474	604 (2.6)	6656	168 (2.5)	9033	274 (3.0)	7785	162 (2.1)	<0.001*	ns	ns	*
Female	36 069	1612 (4.5)	11 484	404 (3.5)	15 310	914 (6.0)	9275	294 (3.2)	<0.001*	*	ns	*
Lifetime prevalence of SD	59 543	5087 (8.5)	18 140	1366 (7.5)	24 343	2478 (10.2)	17 060	1243 (7.3)	<0.001*	*	ns	*
Male	23 474	1684 (7.2)	6656	445 (6.7)	9033	725 (8.0)	7785	514 (6.6)	<0.001*	*	ns	*
Female	36 069	3403 (9.4)	11 484	921 (8.0)	15 310	1753 (11.5)	9275	729 (7.9)	<0.001*	*	ns	*
**Ecuador**												
Point prevalence of SD	3582	112 (3.1)	1200	30 (2.5)	1264	45 (3.6)	1118	37 (3.3)	0.292	ns	ns	ns
Male	1435	38 (2.6)	454	8 (1.8)	497	12 (2.4)	484	18 (3.7)	0.162	ns	ns	ns
Female	2147	74 (3.4)	746	22 (2.9)	767	33 (4.3)	634	19 (3.0)	0.269	ns	ns	ns
Lifetime prevalence of SD	3582	393 (11.0)	1200	127 (10.6)	1264	139 (11.0)	1118	127 (11.4)	0.836	ns	ns	ns
Male	1435	150 (10.5)	454	45 (9.9)	497	46 (9.3)	484	59 (12.2)	0.292	ns	ns	ns
Female	2147	243 (11.3)	746	82 (11.0)	767	93 (12.1)	634	68 (10.7)	0.671	ns	ns	ns
**Germany**												
Point prevalence of SD	3541	200 (5.6)	1242	51 (4.1)	1104	126 (11.4)	1195	23 (1.9)	<0.001*	*	*	*
Male	1184	47 (4.0)	270	13 (4.8)	269	25 (9.3)	645	9 (1.4)	<0.001*	*	ns	*
Female	2357	153 (6.5)	972	38 (3.9)	835	101 (12.1)	550	14 (2.5)	<0.001*	*	ns	*
Lifetime prevalence of SD	3174	367 (10.4)	1242	133 (10.7)	1104	167 (15.1)	1195	67 (5.6)	<0.001*	*	*	*
Male	1184	94 (7.9)	270	30 (11.1)	269	34 (12.6)	645	30 (4.7)	<0.001*	ns	*	*
Female	2357	273 (11.6)	972	103 (10.6)	835	133 (15.9)	550	37 (6.7)	<0.001*	*	*	*
**India**												
Point prevalence of SD	3092	50 (1.6)	1004	15 (1.5)	1086	30 (2.8)	1002	5 (0.5)	<0.001*	ns	ns	*
Male	1208	20 (1.7)	384	7 (1.8)	377	11 (2.9)	447	2 (0.4)	0.021*	ns	ns	*
Female	1884	30 (1.6)	620	8 (1.3)	709	19 (2.7)	555	3 (0.5)	0.008*	ns	ns	*
Lifetime prevalence of SD	3092	73 (2.4)	1004	27 (2.7)	1086	40 (3.7)	1002	6 (0.6)	<0.001*	ns	*	*
Male	1208	29 (2.4)	384	11 (2.9)	377	15 (4.0)	447	3 (0.7)	0.007*	ns	*	*
Female	1884	44 (2.3)	620	16 (2.6)	709	25 (3.5)	555	3 (0.5)	0.002*	ns	*	*
**Iran**												
Point prevalence of SD	3253	19 (0.6)	1031	6 (0.6)	1222	11 (0.2)	1000	2 (0.2)	0.098	ns	ns	ns
Male	1489	7 (0.5)	516	3 (0.6)	521	3 (0.6)	452	1 (0.2)	0.651	ns	ns	ns
Female	1764	12 (0.7)	515	3 (0.6)	701	8 (1.1)	548	1 (0.2)	0.117	ns	ns	ns
Lifetime prevalence of SD	3253	56 (1.7)	1031	14 (1.4)	1222	32 (2.6)	1000	10 (1.0)	0.008*	ns	ns	*
Male	1489	21 (1.4)	516	5 (1.0)	521	10 (1.9)	452	6 (1.3)	0.424	ns	ns	ns
Female	1764	35 (2.0)	515	9 (1.7)	701	22 (3.1)	548	4 (0.7)	0.009*	ns	ns	*
**Kuwait**												
Point prevalence of SD	3556	100 (2.8)	1045	18 (1.7)	1466	56 (3.8)	1045	26 (2.5)	0.006*	*	ns	ns
Male	1624	32 (2.0)	427	7 (1.6)	630	12 (1.9)	567	13 (2.3)	0.755	ns	ns	ns
Female	1932	68 (3.5)	618	11 (1.8)	836	44 (5.3)	478	13 (2.7)	0.001*	*	ns	ns
Lifetime prevalence of SD	3556	209 (5.9)	1045	49 (4.7)	1466	110 (7.5)	1045	50 (4.8)	0.003*	*	ns	*
Male	1624	71 (4.4)	427	22 (5.2)	630	28 (4.4)	567	21 (3.7)	0.539	ns	ns	ns
Female	1932	138 (7.1)	618	27 (4.4)	836	82 (9.8)	478	29 (6.1)	<0.001*	*	ns	ns
**Oman**												
Point prevalence of SD	7106	101 (1.4)	2014	20 (1.0)	3001	80 (2.7)	2064	1 (0.1)	<0.001*	*	ns	*
Male	3389	14 (0.4)	949	5 (0.5)	1280	8 (0.6)	1160	1 (0.1)	0.095	ns	ns	ns
Female	3717	87 (2.3)	1092	15 (1.4)	1721	72 (4.2)	904	0 (0.0)	<0.001*	*	ns	ns
Lifetime prevalence of SD	7106	191 (2.7)	2041	41 (2.0)	3001	147 (4.9)	2064	3 (0.1)	<0.001*	*	ns	*
Male	3389	45 (1.3)	949	13 (1.4)	1280	31 (2.4)	1160	1 (0.1)	<0.001*	ns	*	*
Female	3717	146 (3.9)	1092	28 (2.6)	1721	116 (6.7)	904	2 (0.2)	<0.001*	*	*	*
**Peru**												
Point prevalence of SD	6447	143 (2.2)	2045	66 (3.2)	2398	36 (1.5)	2004	41 (2.0)	<0.001*	*	ns	ns

	All age		Age 18-24 years		Age 25-60 years		Age >60 years		*P* value	***P* value** between group		
	**Total**	**n (%)**	**Total**	**n (%)**	**Total**	**n (%)**	**Total**	**n (%)**		18-24 years and 25-60 years	18-24 years and >60 years	25-60 years and >60 years
Male	3305	75 (2.3)	927	39 (4.2)	1259	14 (1.1)	1119	22 (2.0)	<0.001*	*	*	ns
Female	3142	68 (2.2)	1118	27 (2.4)	1139	22 (1.9)	885	19 (2.1)	0.732	ns	ns	ns
Lifetime prevalence of SD	6447	563 (8.7)	2045	187 (9.1)	2398	182 (7.6)	2004	194 (9.7)	0.036*	ns	ns	*
Male	3305	396 (12.0)	927	130 (14.0)	1259	99 (7.9)	1119	167 (14.9)	<0.001*	*	ns	*
Female	3142	167 (5.3)	1118	57 (5.1)	1139	83 (7.3)	885	27 (3.1)	<0.001*	ns	ns	*
**Poland**												
Point prevalence of SD	12 084	454 (3.8)	3441	120 (3.5)	6196	304 (4.9)	2447	30 (1.2)	<0.001*	*	*	*
Male	4161	100 (2.4)	1040	23 (2.2)	2062	69 (3.3)	1059	8 (0.8)	<0.001*	ns	*	*
Female	7923	354 (4.5)	2401	97 (4.0)	4134	235 (5.7)	1388	22 (1.6)	<0.001*	*	*	*
Lifetime prevalence of SD	12 084	792 (6.6)	3441	203 (5.9)	6196	520 (8.4)	2447	69 (2.8)	<0.001*	*	*	*
Male	4161	179 (4.3)	1040	37 (3.6)	2062	122 (5.9)	1059	20 (1.9)	<0.001*	*	ns	*
Female	7923	613 (7.7)	2401	166 (6.9)	4134	398 (9.6)	1388	49 (3.5)	<0.001*	*	*	*
**Portugal**												
Point prevalence of SD	4254	218 (5.1)	1001	53 (5.3)	2253	138 (6.1)	1000	27 (2.7)	<0.001*	ns	*	*
Male	1282	43 (3.4)	301	11 (3.7)	614	24 (3.9)	367	8 (2.2)	0.328	ns	ns	ns
Female	2972	175 (5.9)	700	42 (6.0)	1639	114 (7.0)	633	19 (3.0)	0.002*	ns	*	*
Lifetime prevalence of SD	4254	513 (12.1)	1001	117 (11.7)	2253	304 (13.5)	1000	92 (9.2)	0.002*	ns	ns	*
Male	1282	108 (8.4)	301	28 (9.3)	614	54 (8.8)	367	26 (7.1)	0.532	ns	ns	ns
Female	2972	405 (13.6)	700	89 (12.7)	1639	250 (15.3)	633	66 (10.4)	0.008*	ns	ns	*
**Russia**												
Point prevalence of SD	6335	355 (5.6)	2036	90 (4.4)	2265	154 (6.8)	2034	111 (5.5)	0.003*	*	ns	ns
Male	2157	94 (4.4)	628	21 (3.3)	798	38 (4.8)	731	35 (4.8)	0.335	ns	ns	ns
Female	4178	261 (6.2)	1408	69 (4.9)	1467	116 (7.9)	1303	76 (5.8)	0.003*	*	ns	ns
Lifetime prevalence of SD	6335	961 (15.2)	2036	222 (10.9)	2265	435 (19.2)	2034	304 (14.9)	<0.001*	*	*	*
Male	2157	307 (14.2)	628	44 (7.0)	798	163 (20.4)	731	100 (13.7)	<0.001*	*	*	*
Female	4178	654 (15.7)	1408	178 (12.6)	1467	272 (18.5)	1303	204 (15.7)	<0.001*	*	ns	ns
**Saudi Arabia**												
Point prevalence of SD	3046	124 (4.1)	1008	25 (2.5)	1003	62 (6.2)	1035	37 (3.6)	<0.001*	*	ns	*
Male	1293	46 (3.6)	449	15 (3.3)	404	20 (5.0)	440	11 (2.5)	0.151	ns	ns	ns
Female	1753	78 (4.4)	559	10 (1.8)	599	42 (7.0)	595	26 (4.4)	<0.001*	*	*	ns
Lifetime prevalence of SD	3046	196 (93.6)	1008	62 (6.2)	1003	82 (8.2)	1035	52 (5.0)	0.014*	ns	ns	*
Male	1293	75 (5.8)	449	34 (7.6)	404	27 (6.7)	440	14 (3.2)	0.013*	ns	*	ns
Female	1753	121 (6.9)	559	28 (5.0)	599	55 (9.2)	595	38 (6.4)	0.016*	*	ns	ns
**Thailand**						3						
Point prevalence of SD	3247	340 (10.5)	1046	78 (7.5)	1085	146 (13.5)	1116	116 (10.4)	<0.001*	*	ns	ns
Male	947	88 (9.3)	311	16 (5.1)	322	38 (11.8)	314	34 (10.8)	0.008*	*	*	ns
Female	2300	252 (11.0)	735	62 (8.4)	763	108 (14.2)	802	82 (10.2)	0.001*	*	ns	ns
Lifetime prevalence of SD	3247	773 (23.8)	1046	184 (17.6)	1085	320 (29.5)	1116	269 (24.1)	<0.001*	*	ns	*
Male	947	209 (22.1)	311	46 (14.8)	322	96 (29.8)	314	67 (21.3)	<0.001*	*	ns	*
Female	2300	564 (24.5)	735	138 (18.8)	763	224 (29.4)	802	202 (25.2)	<0.001*	*	*	ns

* Statistical significance at $P < 0.05$

Abbreviations: ns, nonsignificant; SD, symptomatic dermographism

Supplementary Table S7. Comparison of prevalence of symptomatic dermographism by sex across 12 countries

	Both sexes n/Total (%)		Male n/Total (%)		Female n/Total (%)		*P* value
**International**							
Point prevalence of SD	59 543	2216 (3.7)	23 474	604 (2.6)	36 069	1612 (4.5)	<0.001*
18 – 24 years	18 140	572 (3.2)	6656	168 (2.5)	11 484	404 (3.5)	<0.001*
25 – 60 years	24 343	1188 (4.9)	9033	274 (3.0)	15 310	914 (6.0)	<0.001*
Over 60 years	17 060	456 (2.7)	7785	162 (2.1)	9275	294 (3.2)	<0.001*
Lifetime prevalence of SD	59 543	5087 (8.5)	23 474	1684 (4.2)	36 069	3403 (9.4)	<0.001*
18 – 24 years	18 140	1366 (7.5)	6656	445 (6.7)	11 484	921 (8.0)	0.001*
25 – 60 years	24 343	2478 (10.2)	9033	725 (8.0)	15 310	1753 (11.5)	<0.001*
Over 60 years	17 060	1243 (7.3)	7785	514 (6.6)	9275	729 (7.9)	0.002*
**Ecuador**							
Point prevalence of SD	3582	112 (3.1)	1435	38 (2.6)	2147	74 (3.4)	0.178
18 – 24 years	1200	30 (2.5)	454	8 (1.8)	746	22 (2.9)	0.202
25 – 60 years	1264	45 (3.6)	497	12 (2.4)	767	33 (4.3)	0.077
Over 60 years	1118	37 (3.3)	484	18 (3.7)	634	19 (3.0)	0.504
Lifetime prevalence of SD	3582	393 (11.0)	1435	150 (10.5)	2147	243 (11.3)	0.417
18 – 24 years	1200	127 (10.6)	454	45 (9.9)	746	82 (11.0)	0.555
25 – 60 years	1264	139 (11.0)	497	46 (9.3)	767	93 (12.1)	0.111
Over 60 years	1118	127 (11.4)	484	59 (12.2)	634	68 (10.7)	0.444
**Germany**							
Point prevalence of SD	3541	200 (5.6)	1184	47 (4.0)	2357	153 (6.5)	0.002*
18 – 24 years	1242	51 (4.1)	270	13 (4.8)	972	38 (3.9)	0.507
25 – 60 years	1104	126 (11.4)	269	25 (9.3)	835	101 (12.1)	0.209
Over 60 years	1195	23 (1.9)	645	9 (1.4)	550	14 (2.5)	0.149
Lifetime prevalence of SD	3541	367 (10.4)	1184	94 (7.9)	2357	273 (11.6)	0.001*
18 – 24 years	1242	133 (10.7)	270	30 (11.1)	972	103 (10.6)	0.809
25 – 60 years	1104	167 (15.1)	269	34 (12.6)	835	133 (15.9)	0.190
Over 60 years	1195	67 (5.6)	645	30 (4.7)	550	37 (6.7)	0.120
**India**							
Point prevalence of SD	3092	50 (1.6)	1208	20 (1.7)	1884	30 (1.6)	0.892
18 – 24 years	1004	15 (1.5)	384	7 (1.8)	620	8 (1.3)	0.499
25 – 60 years	1086	30 (2.8)	377	11 (2.9)	709	19 (2.7)	0.820
Over 60 years	1002	5 (0.5)	447	2 (0.4)	555	3 (0.5)	1.000
Lifetime prevalence of SD	3092	73 (2.4)	1208	29 (2.4)	1884	44 (2.3)	0.907
18 – 24 years	1004	27 (2.7)	384	11 (2.9)	620	16 (2.6)	0.787
25 – 60 years	1086	40 (3.7)	377	15 (4.0)	709	25 (3.5)	0.706
Over 60 years	1002	6 (0.6)	447	3 (0.7)	555	3 (0.5)	1.000
**Iran**							
Point prevalence of SD	3253	19 (0.6)	1489	7 (0.5)	1764	12 (0.7)	0.433
18 – 24 years	1031	6 (0.6)	516	3 (0.6)	515	3 (0.6)	1.000
25 – 60 years	1222	11 (0.2)	521	3 (0.6)	701	8 (1.1)	0.371
Over 60 years	1000	2 (0.2)	452	1 (0.2)	548	1 (0.2)	1.000
Lifetime prevalence of SD	3253	56 (1.7)	1489	21 (1.4)	1764	35 (2.0)	0.210
18 – 24 years	1031	14 (1.4)	516	5 (1.0)	515	9 (1.7)	0.280
25 – 60 years	1222	32 (2.6)	521	10 (1.9)	701	22 (3.1)	0.187
Over 60 years	1000	10 (1.0)	452	6 (1.3)	548	4 (0.7)	0.361
**Kuwait**							
Point prevalence of SD	3556	100 (2.8)	1624	32 (2.0)	1932	68 (3.5)	0.005*
18 – 24 years	1045	18 (1.7)	427	7 (1.6)	618	11 (1.8)	0.864
25 – 60 years	1466	56 (3.8)	630	12 (1.9)	836	44 (5.3)	<0.001*
Over 60 years	1045	26 (2.5)	567	13 (2.3)	478	13 (2.7)	0.659

	Both sexes n/Total (%)		Male n/Total (%)		Female n/Total (%)		*P* value
Lifetime prevalence of SD	3556	209 (5.9)	1624	71 (4.4)	1932	138 (7.1)	<0.001*
18 – 24 years	1045	49 (4.7)	427	22 (5.2)	618	27 (4.4)	0.556
25 – 60 years	1466	110 (7.5)	630	28 (4.4)	836	82 (9.8)	<0.001*
Over 60 years	1045	50 (4.8)	567	21 (3.7)	478	29 (6.1)	0.075
**Oman**							
Point prevalence of SD	7106	101 (1.4)	3389	14 (0.4)	3717	87 (2.3)	<0.001*
18 – 24 years	2014	20 (1.0)	949	5 (0.5)	1092	15 (1.4)	0.053
25 – 60 years	3001	80 (2.7)	1280	8 (0.6)	1721	72 (4.2)	<0.001*
Over 60 years	2064	1 (0.1)	1160	1 (0.1)	904	0 (0.0)	1.000
Lifetime prevalence of SD	7106	191 (2.7)	3389	45 (1.3)	3717	146 (3.9)	<0.001*
18 – 24 years	2041	41 (2.0)	949	13 (1.4)	1092	28 (2.6)	0.055
25 – 60 years	3001	147 (4.9)	1280	31 (2.4)	1721	116 (6.7)	<0.001*
Over 60 years	2064	3 (0.1)	1160	1 (0.1)	904	2 (0.2)	0.585
**Peru**							
Point prevalence of SD	6447	143 (2.2)	3305	75 (2.3)	3142	68 (2.2)	0.775
18 – 24 years	2045	66 (3.2)	927	39 (4.2)	1118	27 (2.4)	0.022*
25 – 60 years	2398	36 (1.5)	1259	14 (1.1)	1139	22 (1.9)	0.099
Over 60 years	2004	41 (2.0)	1119	22 (2.0)	885	19 (2.1)	0.776
Lifetime prevalence of SD	6447	563 (8.7)	3305	396 (12.0)	3142	167 (5.3)	<0.001*
18 – 24 years	2045	187 (9.1)	927	130 (14.0)	1118	57 (5.1)	<0.001*
25 – 60 years	2398	182 (7.6)	1259	99 (7.9)	1139	83 (7.3)	0.595
Over 60 years	2004	194 (9.7)	1119	167 (14.9)	885	27 (3.1)	<0.001*
**Poland**							
Point prevalence of SD	12 084	454 (3.8)	4161	100 (2.4)	7923	354 (4.5)	<0.001*
18 – 24 years	3441	120 (3.5)	1040	23 (2.2)	2401	97 (4.0)	0.007*
25 – 60 years	6196	304 (4.9)	2062	69 (3.3)	4134	235 (5.7)	<0.001*
Over 60 years	2447	30 (1.2)	1059	8 (0.8)	1388	22 (1.6)	0.065
Lifetime prevalence of SD	12 084	792 (6.6)	4161	179 (4.3)	7923	613 (7.7)	<0.001*
18 – 24 years	3441	203 (5.9)	1040	37 (3.6)	2401	166 (6.9)	<0.001*
25 – 60 years	6196	520 (8.4)	2062	122 (5.9)	4134	398 (9.6)	<0.001*
Over 60 years	2447	69 (2.8)	1059	20 (1.9)	1388	49 (3.5)	0.015*
**Portugal**							
Point prevalence of SD	4254	218 (5.1)	1282	43 (3.4)	2972	175 (5.9)	0.001*
18 – 24 years	1001	53 (5.3)	301	11 (3.7)	700	42 (6.0)	0.129
25 – 60 years	2253	138 (6.1)	614	24 (3.9)	1639	114 (7.0)	0.007*
Over 60 years	1000	27 (2.7)	367	8 (2.2)	633	19 (3.0)	0.440
Lifetime prevalence of SD	4254	513 (12.1)	1282	108 (8.4)	2972	405 (13.6)	<0.001*
18 – 24 years	1001	117 (11.7)	301	28 (9.3)	700	89 (12.7)	0.123
25 – 60 years	2253	304 (13.5)	614	54 (8.8)	1639	250 (15.3)	<0.001*
Over 60 years	1000	92 (9.2)	367	26 (7.1)	633	66 (10.4)	0.078
**Russia**							
Point prevalence of SD	6335	355 (5.6)	2157	94 (4.4)	4178	261 (6.2)	0.002*
18 – 24 years	2036	90 (4.4)	628	21 (3.3)	1408	69 (4.9)	0.115
25 – 60 years	2265	154 (6.8)	798	38 (4.8)	1467	116 (7.9)	0.005*
Over 60 years	2034	111 (5.5)	731	35 (4.8)	1303	76 (5.8)	0.320
Lifetime prevalence of SD	6335	961 (15.2)	2157	307 (14.2)	4178	654 (15.7)	0.135
18 – 24 years	2036	222 (10.9)	628	44 (7.0)	1408	178 (12.6)	<0.001*
25 – 60 years	2265	435 (19.2)	798	163 (20.4)	1467	272 (18.5)	0.277
Over 60 years	2034	304 (14.9)	731	100 (13.7)	1303	204 (15.7)	0.230
**Saudi Arabia**							
Point prevalence of SD	3046	124 (4.1)	1293	46 (3.6)	1753	78 (4.4)	0.218
18 – 24 years	1008	25 (2.5)	449	15 (3.3)	559	10 (1.8)	0.115
25 – 60 years	1003	62 (6.2)	404	20 (5.0)	599	42 (7.0)	0.184

	Both sexes n/Total (%)		Male n/Total (%)		Female n/Total (%)		*P* value
Over 60 years	1035	37 (3.6)	440	11 (2.5)	595	26 (4.4)	0.109
Lifetime prevalence of SD	3046	196 (93.6)	1293	75 (5.8)	1753	121 (6.9)	0.221
18 – 24 years	1008	62 (6.2)	449	34 (7.6)	559	28 (5.0)	0.092
25 – 60 years	1003	82 (8.2)	404	27 (6.7)	599	55 (9.2)	0.157
Over 60 years	1035	52 (5.0)	440	14 (3.2)	595	38 (6.4)	0.020*
**Thailand**							
Point prevalence of SD	3247	340 (10.5)	947	88 (9.3)	2300	252 (11.0)	0.159
18 – 24 years	1046	78 (7.5)	311	16 (5.1)	735	62 (8.4)	0.064
25 – 60 years	1085	146 (13.5)	322	38 (11.8)	763	108 (14.2)	0.299
Over 60 years	1116	116 (10.4)	314	34 (10.8)	802	82 (10.2)	0.766
Lifetime prevalence of SD	3247	773 (23.8)	947	209 (22.1)	2300	564 (24.5)	0.136
18 – 24 years	1046	184 (17.6)	311	46 (14.8)	735	138 (18.8)	0.122
25 – 60 years	1085	320 (29.5)	322	96 (29.8)	763	224 (29.4)	0.880
Over 60 years	1116	269 (24.1)	314	67 (21.3)	802	202 (25.2)	0.176

* Statistical significance at $P < 0.05$

Abbreviations: SD, symptomatic dermographism

Supplementary Figure S1A. The flow diagram of questions in the questionnaire
After accessing the Google Forms PREVALENCE-D questionnaire, each participant was asked for his/her willingness to participate in the study. Firstly, demographic data of each participant was collected. Subsequently, the presence of physiological red dermographism, simple urticarial dermographism, and symptomatic dermographism was determined. For participants exhibiting symptomatic dermographism, further details regarding its characteristics were gathered.

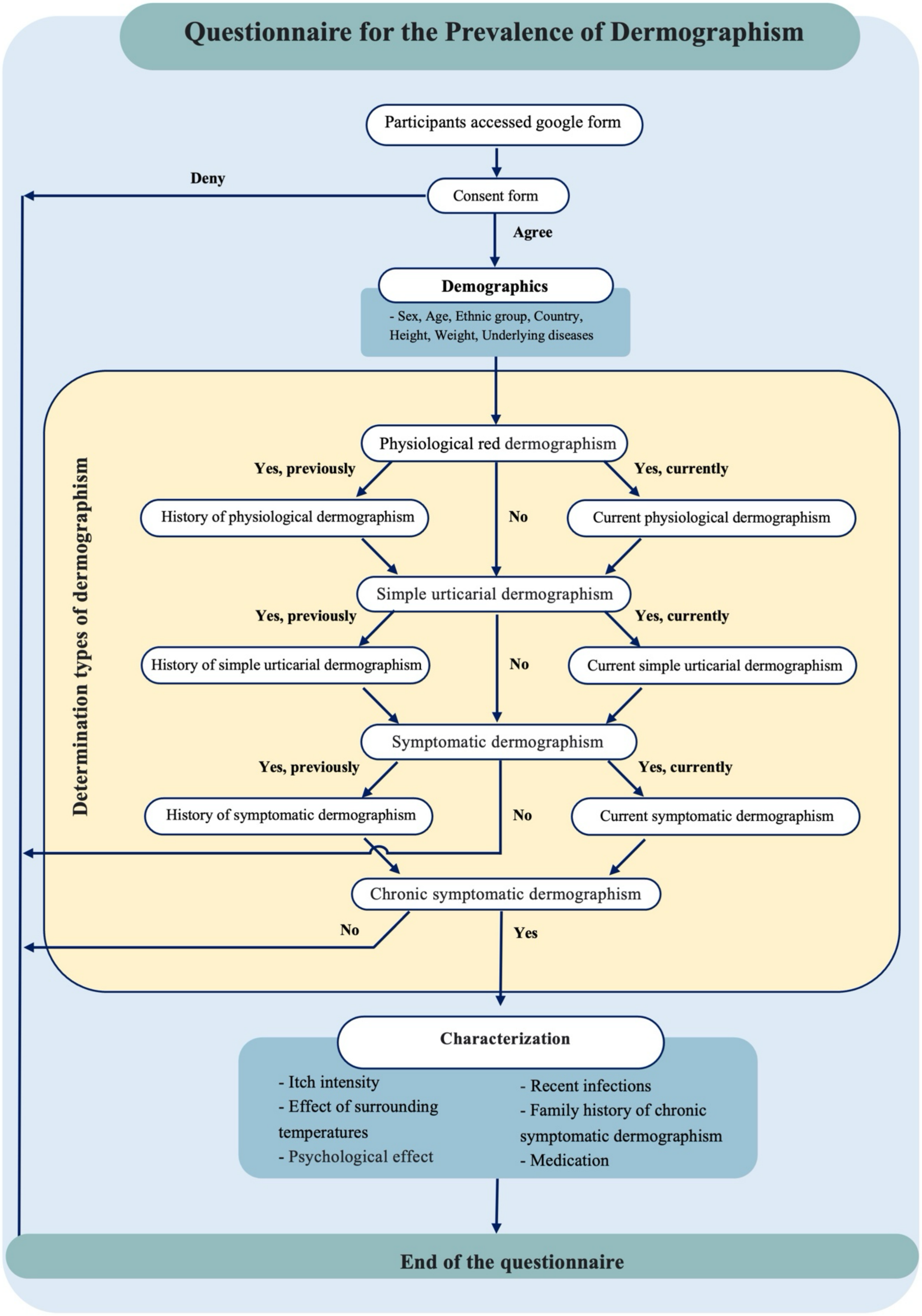

Supplementary Figure S1B. The flow diagram of questions in the questionnaire

Questionnaire for the Prevalence of Dermographism

This survey is conducted by the research team of Urticaria Centers of Reference and Excellence

This survey aims to know the prevalence of dermographism in general population, the impact of this problem and help healthcare workers improve the quality of care for this condition in the future.

This survey requires the response from people with or without dermographism to know the prevalence in the general population. You can choose whether or not to complete this survey. No identifiable personal information will be asked so no one will know who you are. Your responses to the survey will be analyzed together with those from other people who completed the survey as well and will be presented as an overall summary of information.

Dermographism is a transient reaction of the skin that occurs when the skin is scratched or rubbed with a blunt object. Each lesion of dermographism happens for a short time and recovery to normal skin.

When this reaction causes a redness that is not raised and is not itchy, it is called red demographism (Photo 1). This dermographism is normal and not pathological.

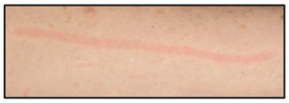

Photo 1. Red dermographism (not raised and not itchy)

In some people, when the skin is scratched or rubbed, a reaction occurs in which skin is raised (you can feel the reaction). This raised line (wheal) is often red at first and then becomes whitish, showing redness of the surrounding skin (Photo 2). This form of dermographism is referred to as urticarial dermographism.

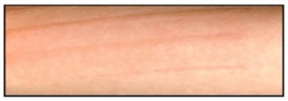

Photo 2. Urticarial dermographism (reddened and raised)

Some people have recurrent urticarial dermographism that itches. This is called chronic symptomatic dermographism. The purpose of this survey is to clarify the frequency of red dermographism, urticarial dermographism and chronic symptomatic dermographism.

This survey only takes a few minutes to complete. The decision to participate in this survey will not have any consequence against your medical care in any way.

- If you do not want to participate, please select "I do not agree to participate".
- If you wish to participate, please select "I agree to participate" which is your consent approval and then respond to the subsequent questions.

I have read and understood this consent form.

☐ I do not agree to participate the survey ***(participant will end the questionnaire)***
☐ I voluntary agree to participate the survey ***(participant will start the questionnaire)***

Part 1. Demographics of the participant

1.1 Gender binary ☐ Male ☐ Female
1.2 Age ………. years

1.3 What ethnic group do you identify with/belong to?

☐ Caucasian ☐ Middle Eastern ☐ African
☐ Latino/ Hispanic ☐ East Asian ☐ South Asian (including Indian)
☐ Southeast Asian ☐ Caribbean ☐ Others (please specify) ………

1.4 Which country do you live in? (please specify) ……………………………

1.5 My height (please choose unit to specify) in Centimeters or Feet and Inches
For Centimeters (please go to 1.5.1)
1.5.1 Height (please specify) ……………………………

For Feet and Inches (please go to 1.5.2 and 1.5.3)
1.5.2 Height (please specify Feet) ………………
1.5.3 Height (please specify Inches) ……………

1.6 My body weight (please choose unit to specify) in Kilograms or Pounds
1.6.1 Body weight (please specify) ………………………

1.7 Do you have any /disease (s) that require (s) continuous medication (s)?

☐ No
☐ Yes (please choose the box that is applicable to you)
☐ Allergic rhinitis (frequent nasal congestion or stuffy nose)
☐ Asthma
☐ Atopic dermatitis (chronic relapsing red and itchy skin rashes on flexural areas or skin folds and elsewhere together with dry skin)
☐ Chronic hives with or without eyelid/lip swelling (angioedema)
☐ Diabetes Mellitus ☐ Hypertension
☐ Dyslipidemia
☐ Cardiovascular disease ☐ Cerebrovascular disease
☐ Liver disease ☐ Renal disease
☐ Thyroid disease ☐ Cancer
☐ Other skin diseases (please specify) …………………………..
☐ Others (please specify) …………………………..

Part 2. Determination if you have different types of dermographism

2.1 Have you ever had a red dermographism (a reddened flat line on a particular part of the skin where you scratched it or was rubbed against a hard object, such as the edge of a table similar to Photo 3?

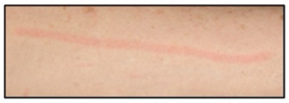

Photo 3. Red dermographism

☐ Currently yes (please respond to 2.1.1 and 2.1.2)
☐ Yes, in the past but not anymore (please respond to 2.1.1 and 2.1.2)
☐ No, never (please go to 2.2)

2.1.1 Has your red dermographism only occurred after a hard scratch?
☐ Yes ☐ No (it can even occur after a light scratch)
2.1.2 Does your red dermographism itch as well?
☐ Yes ☐ No

2.2 Have you ever had an urticarial dermographism (a reddened raised line on a particular part of the skin where you scratched it or was rubbed against a hard object, such as the edge of a table similar to Photo 4?

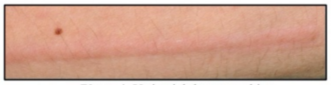

Photo 4. Urticarial dermographism

☐ Currently yes (please respond to 2.2.1 and 2.2.2)
☐ Yes, in the past but not anymore (please respond to 2.2.1 and 2.2.2)
☐ No, never (please go to 2.3)

2.2.1 Has your urticarial dermographism only occurred after a hard scratch?
☐ Yes ☐ No (it can even occur after a light scratch)
2.2.2 Does your urticarial dermographism itch as well?
☐ Yes ☐ No

2.3 Do you have recurrent urticarial dermographism called chronic symptomatic dermographism (a reddened raised line as in 2.2 but that appears often)?

☐ Currently yes (please respond to 2.3.1 and 2.3.2)
☐ Yes, in the past but not anymore (please respond to 2.3.1 and 2.3.2)
☐ No ***(participants will end the questionnaire)***

2.3.1 Has your recurrent urticarial dermographism only occurred after a hard scratch?
☐ Yes ☐ No (it can even occur after a light scratch)
2.3.2 Does your recurrent urticarial dermographism itch as well?
☐ Yes ☐ No ***(participants will end the questionnaire)***

Part 3. Characteristics of chronic symptomatic dermographism

3.1 What is the average itch intensity of your chronic symptomatic dermographism?

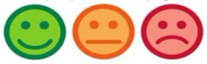

☐ Mild itch = present but not annoying or troublesome
☐ Moderate itch = troublesome but does not interfere with normal daily activity or sleep
☐ Severe itch = sufficient troublesome to interfere with normal daily activity or sleep

3.2 Do changes in surrounding temperatures affect your chronic symptomatic dermographism?

☐ It gets better when it is cold
☐ It gets worse when it is cold
☐ It gets better when it is warm
☐ It gets worse when it is warm
☐ Not associated

3.3 Does your chronic symptomatic dermographism cause psychological stress, anxiety, depression, isolation, fear or mood lability?

☐ No
☐ Yes, specify …………………………………

3.4 Have you had an infection during the last year?

☐ No
☐ Yes (you can mark more than one answer if it applies)

☐ Skin infection ☐ Parasitic infections such as scabies, worms or bedbugs
☐ Tinea or ringworm infection ☐ Hair infection such as lice
☐ Bowel infection ☐ Others (specify) …………….

3.5 Does anyone in your family (parents/siblings/children) have chronic symptomatic dermographism?

☐ No
☐ Yes, specific who ……………………
☐ Unknown

3.6 Do you take any medicines to control your chronic symptomatic dermographism? (you can mark more than one answer if it applies)

☐ Yes, H1-antihistamines (e.g. cetirizine, fexofenadine, loratadine…)
☐ Yes, H2-antihistamines (e.g. famotidine…)
☐ Yes, leukotriene modifying agents (e.g. montelukast…)
☐ Yes, Omalizumab (Xolair)
☐ Yes, don't know the name of the drug (s)
☐ Yes, other drugs, please specify ……………………
☐ No

Thank you for taking your time to complete the survey.

(End of questionnaire and click submit)

Supplementary Figure S2. Supplementary methods for calculation of international sex- and age- adjusted prevalence rate

Supplementary methods

Calculation of international sex- and age-adjusted prevalence rate

Stratum (h)	Country	Sex	Age group	Population: No. of pop'n (N_h)	Population: Pop'n weight ($W_h = N_h / N$)	Sample: No. of sample (n_h)	Sample: Sample weight ($w_h = n_h/n$)	Sample: No. of sample with disease (x_h)	Sample: Proportion of disease ($p_h = x_h/n_h$)
1	1	M	1	N_1	$W_1 = N_1/N$	n_1	$w_1 = n_1/n$	x_1	$p_1 = x_1/n_1$
2			2	N_2	$W_2 = N_2/N$	n_2	$w_2 = n_2/n$	x_2	$p_2 = x_2/n_2$
3			3	N_3	$W_3 = N_3/N$	n_3	$w_3 = n_3/n$	x_3	$p_3 = x_3/n_3$
4		F	1						
5			2						
6			3						
7	2	M	1						
8			2						
9			3						
..		…	.	…	…	…	…	..	…
67	12	M	1						
68			2						
69			3						
70		F	1						
71			2						
72			3	N_{72}	$W_{72} = N_{72}/N$	n_{72}	$w_{72} = n_{72}/n$	x_{72}	$p_{72} = x_{72}/n_{72}$
Total (Sum)				N	1	n	1		

where

h = Stratum of country (12 countries) by sex (M, F) and age (3 age groups), h = 1 to 72

N_h = Number of population in stratum h

N = Total population size = $N_1 + N_2 + \ldots + N_{72}$

W_h = Population weight = N_h / N, $\Sigma W_h = 1$

n_h = Number of sample in stratum h

n = Total sample size = $n_1 + n_2 + \ldots + n_{72}$

w_h = Sample weight = n_h / n, $\Sigma w_h = 1$

x_h = Number of sample with disease in stratum h

p_h = (Sample) Proportion of disease in stratum h = x_h / n_h

Suppose the disease of interest is symptomatic dermographism (SD) and the purpose is to estimate the international prevalence of SD. Since the prevalence of SD depends on sex and age and the distribution of sex and age in the sample is not the same as that in the population (i.e., $w_h \neq W_h$), the sex- and age-adjusted international prevalence needs to be computed as shown below.

p_w = Sex- and age-adjusted prevalence = $\Sigma\, p_h * W_h$

Variance(p_w) = $\sum W_h^2 p_h(1 - p_h)/n_h$

SE(p_w) = $\sqrt{Variance(p_w)}$

95% confidence interval of P_w = $p_w \pm 1.96*SE(p_w)$ = l, u (l = lower, u = upper)

Thus, as a rate (%)

Sex- and age-adjusted prevalence rate = $100*p_w$

95% confidence interval of adjusted prevalence rate = (100*l, 100*u)

REFERENCE

1. Cotton TF, Slade JG, Lewis TS. *Observations upon dermatographism with special reference to the contractile power of capillaries*. [Heart]; 1917:pp227-48

2. Lewis T. Vascular reactions of the skin to injury. Part 1. Reaction to stroking ; urticaria factitia. *Heart*. 1924 1924;11:119-140.

3. Walzer A. Urticaria III. Experimental urticaria factitia. *Arch Derm Syphilol*. 1928;18:868-86.

4. Fisher AA, Schwartz S. Low incidence of dermographism in subacute and chronic urticaria. *AMA Arch Derm Syphilol*. Nov 1953;68(5):553-5. doi:10.1001/archderm.1953.01540110075011

5. Ebken RK, Bauschard FA, Levine MI. Dermographism: its definition, demonstration, and prevalence. *J Allergy*. Jun 1968;41(6):338-43. doi:10.1016/0021-8707(68)90076-2

6. Kirby JD, Matthews CN, James J, Duncan EH, Warin RP. The incidence and other aspects of factitious wealing (dermographism). *Br J Dermatol*. Oct 1971;85(4):331-5. doi:10.1111/j.1365-2133.1971.tb14027.x

7. Margolis CF, Estes SA. Symptomatic dermographism. *J Fam Pract*. Dec 1981;13(7):993-5.

8. Breathnach SM, Allen R, Ward AM, Greaves MW. Symptomatic dermographism: natural history, clinical features laboratory investigations and response to therapy. *Clin Exp Dermatol*. Sep 1983;8(5):463-76. doi:10.1111/j.1365-2230.1983.tb01814.x

9. Matthews CN, Kirby JD, James J, Warin RP. Dermographism: reduction in weal size by chlorpheniramine and hydroxyzine. *Br J Dermatol*. Mar 1973;88(3):279-82. doi:10.1111/j.1365-2133.1973.tb07548.x

10. Wong RC, Fairley JA, Ellis CN. Dermographism: a review. *J Am Acad Dermatol*. Oct 1984;11(4 Pt 1):643-52. doi:10.1016/s0190-9622(84)70222-2

11. Kontou-Fili K, Borici-Mazi R, Kapp A, Matjevic LJ, Mitchel FB. Physical urticaria: classification and diagnostic guidelines. An EAACI position paper. *Allergy*. May 1997;52(5):504-13. doi:10.1111/j.1398-9995.1997.tb02593.x

12. Martorell A, Sanz J, Ortiz M, et al. Prevalence of dermographism in children. *J Investig Allergol Clin Immunol*. May-Jun 2000;10(3):166-9.

13. Schoepke N, Młynek A, Weller K, Church MK, Maurer M. Symptomatic dermographism: an inadequately described disease. *J Eur Acad Dermatol Venereol*. Apr 2015;29(4):708-12. doi:10.1111/jdv.12661

14. Sánchez-Borges M, González-Aveledo L, Caballero-Fonseca F, Capriles-Hulett A. Review of Physical Urticarias and Testing Methods. *Curr Allergy Asthma Rep*. Aug 2017;17(8):51. doi:10.1007/s11882-017-0722-1

15. Seo JH, Kwon JW. Epidemiology of urticaria including physical urticaria and angioedema in Korea. *Korean J Intern Med*. Mar 2019;34(2):418-425. doi:10.3904/kjim.2017.203

16. Gu X, Xiao Y, Li S, et al. Air pollution and meteorological factors are associated with dermographism: a population-based study in college students. *J Eur Acad Dermatol Venereol*. Dec 2021;35(12):e920-e921. doi:10.1111/jdv.17586

17. Li J, Mao D, Liu S, et al. Epidemiology of urticaria in China: a population-based study. *Chin Med J (Engl)*. Jun 5 2022;135(11):1369-1375. doi:10.1097/CM9.0000000000002172

18. Cai W, Lu C, Li X, et al. Epidemiology of Superficial Fungal Infections in Guangdong, Southern China: A Retrospective Study from 2004 to 2014. *Mycopathologia*. Jun 2016;181(5-6):387-95. doi:10.1007/s11046-016-9986-6

19. United Nations DoEaSA, Population Division (2024). World Population Prospects 2024, Online Edition.
